# Estimated Clinical Outcomes and Cost-effectiveness Associated With Provision of Addiction Treatment in US Primary Care Clinics

**DOI:** 10.1001/jamanetworkopen.2023.7888

**Published:** 2023-04-12

**Authors:** Raagini Jawa, Yjuliana Tin, Samantha Nall, Susan L. Calcaterra, Alexandra Savinkina, Laura R. Marks, Simeon D. Kimmel, Benjamin P. Linas, Joshua A. Barocas

**Affiliations:** 1 Section of General Internal Medicine, Center for Research on Healthcare, University of Pittsburgh School of Medicine, Pittsburgh, Pennsylvania; 2 Section of Infectious Diseases, Boston Medical Center, Boston, Massachusetts; 3 Division of General Internal Medicine, University of Colorado Anschutz Medical Campus, Aurora; 4 Division of Hospital Medicine, University of Colorado Anschutz Medical Campus, Aurora; 5 Department of Epidemiology of Microbial Diseases, Yale School of Public Health, New Haven, Connecticut; 6 Division of Infectious Diseases, School of Medicine, Washington University in St Louis, Missouri; 7 Section of General Internal Medicine, Boston Medical Center, Boston, Massachusetts; 8 Boston University Chobanian and Avedisian School of Medicine, Boston, Massachusetts; 9 Divisions of General Internal Medicine and Infectious Diseases, University of Colorado Anschutz Medical Campus, Aurora

## Abstract

**Importance:**

US primary care practitioners (PCPs) are the largest clinical workforce, but few provide addiction care. Primary care is a practical place to expand addiction services, including buprenorphine and harm reduction kits, yet the clinical outcomes and health care sector costs are unknown.

**Objective:**

To estimate the long-term clinical outcomes, costs, and cost-effectiveness of integrated buprenorphine and harm reduction kits in primary care for people who inject opioids.

**Design, Setting, and Participants:**

In this modeling study, the Reducing Infections Related to Drug Use Cost-Effectiveness (REDUCE) microsimulation model, which tracks serious injection-related infections, overdose, hospitalization, and death, was used to examine the following treatment strategies: (1) PCP services with external referral to addiction care (status quo), (2) PCP services plus onsite buprenorphine prescribing with referral to offsite harm reduction kits (BUP), and (3) PCP services plus onsite buprenorphine prescribing and harm reduction kits (BUP plus HR). Model inputs were derived from clinical trials and observational cohorts, and costs were discounted annually at 3%. The cost-effectiveness was evaluated over a lifetime from the modified health care sector perspective, and sensitivity analyses were performed to address uncertainty. Model simulation began January 1, 2021, and ran for the entire lifetime of the cohort.

**Main Outcomes and Measures:**

Life-years (LYs), hospitalizations, mortality from sequelae (overdose, severe skin and soft tissue infections, and endocarditis), costs, and incremental cost-effectiveness ratios (ICERs).

**Results:**

The simulated cohort included 2.25 million people and reflected the age and gender of US persons who inject opioids. Status quo resulted in 6.56 discounted LYs at a discounted cost of $203 500 per person (95% credible interval, $203 000-$222 000). Each strategy extended discounted life expectancy: BUP by 0.16 years and BUP plus HR by 0.17 years. Compared with status quo, BUP plus HR reduced sequelae-related mortality by 33%. The mean discounted lifetime cost per person of BUP and BUP plus HR were more than that of the status quo strategy. The dominating strategy was BUP plus HR. Compared with status quo, BUP plus HR was cost-effective (ICER, $34 400 per LY). During a 5-year time horizon, BUP plus HR cost an individual PCP practice approximately $13 000.

**Conclusions and Relevance:**

This modeling study of integrated addiction service in primary care found improved clinical outcomes and modestly increased costs. The integration of addiction service into primary care practices should be a health care system priority.

## Key Points

**Question:**

What are the estimated clinical outcomes, costs, and cost-effectiveness of integrating buprenorphine and harm reduction kits into primary care for people who inject opioids?

**Findings:**

In this decision analytical model using the Reducing Infections Related to Drug Use Cost-Effectiveness model, integrating buprenorphine alone extended discounted life expectancy by 0.16 years and buprenorphine combined with harm reduction kits extended discounted life expectancy by 0.17 years. Compared with the status quo, buprenorphine and harm reduction kits reduced drug use–related mortality by 33% and was cost-effective.

**Meaning:**

These findings suggest that integrating buprenorphine and harm reduction kits into primary care may improve clinical outcomes, modestly increase costs, and be cost-effective to health systems.

## Teaser

This decision analytical model of a simulated representative cohort of people who inject opioids estimates the long-term clinical outcomes, costs, and cost-effectiveness of different strategies of integrating addiction treatment into US primary care.

## Introduction

The US opioid epidemic has led to increasing incidence of opioid-related overdose and injection drug use–related infections, such as infective endocarditis (IE) and severe skin and soft tissue infections (SSTIs).^1,2^ Given the scope of the epidemic, there is a need to rapidly scale up addiction treatment capacity and harm reduction services for opioid use disorder (OUD).

Primary care practitioners (PCPs) are the largest clinical workforce in the US,^3^ but few provide a full spectrum of addiction care on site.^4^ Primary care practices are a practical place to integrate addiction services, where PCPs can prescribe buprenorphine and deliver harm reduction kits. Although PCPs view buprenorphine as effective,^4,5,6^ prescribing has been slow to increase^7,8^ because of the lack of institutional support,^6,9^ low levels of interest in treating OUD because of perceived burden and other factors,^4^ and the need for additional training.^10^ Recent policy changes that removed mandatory training requirements to obtain a buprenorphine waiver^11^ could improve addiction treatment access for patients in all health care settings, including within primary care, where multidisciplinary and coordinated care delivery models have facilitated buprenorphine prescribing.^12,13^

Although prescribing medication for OUD (MOUD) is one way to address OUD and prevent complications, not all patients are ready or willing to initiate pharmacologic treatment. In addition, MOUD does not guarantee abstinence, so there is a need for additional strategies to reduce risk. A harm reduction approach can mitigate risk while acknowledging the conditions that may lead individuals to continue to use substances.^14^ Harm reduction strategies include provision of sterile injection equipment, fentanyl test strips, skin cleaning education, overdose education, and naloxone distribution.^14,15^ Given US regional regulations and legal concerns, harm reduction equipment provision is typically offered in non–health care community settings. However, there is an evolving national priority from the American Rescue Plan to expand access to harm reduction.^16^ Thus, an opportunity exists to colocate these services within primary care, where patients may have existing, trusting relationships with clinicians.^17^

Although integrating buprenorphine and harm reduction kits into primary care may improve clinical outcomes among people who inject opioids, such a shift could include financial tradeoffs between potential immediate costs. We sought to estimate the long-term clinical outcomes, costs, and cost-effectiveness of different strategies of integrating addiction treatment into US primary care.

## Methods

### Analytical Overview

For this decision analytical model, we used a validated Monte Carlo microsimulation model, the Reducing Infection Related to Drug Use Cost-Effectiveness (REDUCE) model, that simulates the natural history of injection opioid use (eMethods in Supplement 1). We used the model to compare the following treatment strategies for integrated addiction care in primary care: (1) standard primary care services, where PCPs refer patients to external addiction care (status quo); (2) standard primary care services plus onsite buprenorphine prescribing with referral to offsite harm reduction kits (BUP); and (3) standard primary care services plus onsite buprenorphine prescribing and harm reduction kit provision (BUP plus HR) (Figure). In all 3 modeled strategies, we assume that patients begin the simulation not treated with MOUD. We also assume that if patients are hospitalized and not receiving MOUD, they undergo inpatient opioid detoxification and are not given MOUD. If they are already receiving MOUD and are hospitalized, then use of that medication is continued on discharge. Race and ethnicity were not factored into this analysis given limitations of the model and limitations of the data used to populate the model. Model simulation began January 1, 2021, and ran for the entire lifetime of the cohort. The study was approved by the University of Colorado Institutional Review Board, which determined the REDUCE model to be non–human subject research. The study followed the Consolidated Health Economic Evaluation Reporting Standards (CHEERS) reporting guideline.

**Figure.**
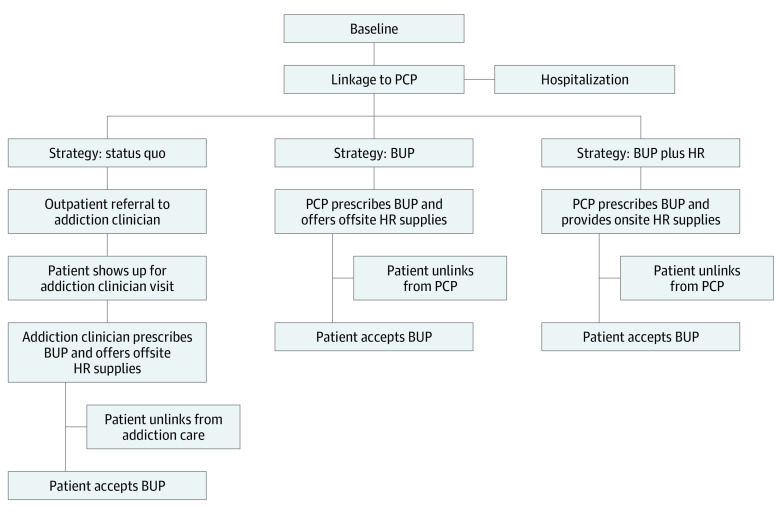
Model Schematic for 3 Strategies Integrating Addiction Care Into Primary Care The 3 strategies were (1) primary care practitioner (PCP) services with external referral to addiction care (status quo), (2) PCP services plus onsite buprenorphine prescribing with referral to offsite harm reduction kits (BUP), and (3) PCP services plus onsite buprenorphine prescribing and harm reduction kits (BUP plus HR).

In the status quo strategy, patients first link to a PCP and are then referred to, and can link to, an offsite addiction specialist for buprenorphine. Patients can obtain harm reduction kits offsite in the community. In the BUP strategy, once patients link to their PCPs, we assume that buprenorphine can be offered at the time during an office visit, but patients will be referred offsite in the community for harm reduction kits. In the BUP plus HR strategy, buprenorphine is offered and harm reduction kits (consisting of a package of 10 sterile syringes with injection preparation equipment, safer smoking kit, and skin hygiene and wound care supplies) are provided at every office visit; thus, no additional linkage is needed for addiction specialist care or offsite harm reduction kits. The Figure outlines the differences among the strategies.

We used the model to estimate long-term outcomes for the cohort beginning in 2021, including percentage of deaths associated with sequelae, averted hospitalizations, life expectancy (measured in life-years [LYs]), mean lifetime costs per person, and incremental cost-effectiveness ratios (ICERs). We projected lifetime medical costs assuming a health care sector perspective, denominated in 2021 US dollars, and applied a 3% discount rate to both costs and LYs.^18^ We also conducted a 5-year budgetary impact analysis from the perspective of PCP practices to determine the cost of offering BUP plus HR.

We calculated ICERs of each treatment strategy as the additional cost per person divided by the LYs gained compared with the next less expensive strategy.^18^ We interpreted ICERs using a willingness-to-pay threshold of $100 000 per LY gained.^18^ We performed deterministic and probabilistic sensitivity analyses to evaluate uncertainty.

### REDUCE Model Overview

#### Model Structure and Simulated Cohort

The REDUCE model has been described in detail elsewhere.^19,20,21^ Briefly, the model is a closed cohort microsimulation, with no new entrants, of the natural history of injection opioid use, including complications such as overdose, infective endocarditis, and severe SSTI (henceforth referred to as sequelae); treatment; and changes in injection behaviors. The model uses a weekly time step and tracks all individuals from model initiation until death.

We simulate the cohort stratified by sex, age, and injection behavior profile, which includes injection frequency (high, low, or not currently injecting drugs), sharing of injection equipment, and sterile injection technique.^21^ We assume that initial age, sex, and injection profile does not differ among the strategies. In each strategy, we model adults 18 years or older who predominantly inject opioids and are not taking MOUD at the start of the simulation.

#### Sequelae of Drug Use and Hospitalization

Only individuals with injection drug use are at risk of sequelae, and the risk is greater with high-frequency drug use than with low-frequency drug use. Individuals who develop sequelae have a probability of being hospitalized and treated for their condition.^22,23^ To account for the sparsity of addiction consultation service in the US, we assumed that hospitalized individuals did not receive a consultation for addiction care services, underwent opioid withdrawal management with buprenorphine or methadone, and did not continue MOUD use after hospital discharge.^24,25,26^ However, if someone was using MOUD before hospitalization, use of this medication would be continued after hospital discharge. Individuals also have a probability of being discharged against medical advice.

#### Outpatient Services

Individuals may link to an outpatient PCP either in the community or after a hospitalization. In the status quo strategy, linkage for individuals who are not taking buprenorphine is first to their PCP with referral to an offsite addiction specialist. If patients present to their addiction specialists, they are offered home-induction buprenorphine, which they can then decide to use. In this strategy, the addiction specialist continues to prescribe buprenorphine until the patient unlinks from care. In both the BUP and BUP plus HR strategies, once a patient links to a PCP, we assume that the PCP has the capacity to initiate and continue to prescribe buprenorphine and that it is offered to all patients who link to care (but not all will accept the offer).^27^ In both the status quo and BUP strategies, we assume that patients are receiving harm reduction kits offsite in the community through a background mechanism. In the BUP plus HR strategy, we assume that naloxone is supplied onsite at the intake visit and that harm reduction kits are provided at every visit.

#### Mortality

Individuals face a risk of death from sequelae as well as age- and sex-related causes (ie, competing causes). We derived the probability of a fatal overdose in each week as the product of the probability of having an overdose based on an individual’s age, sex, injection profile, and the probability of death conditional on having an opioid overdose.

#### Costs

Individuals accrue weekly costs related to disease status and service use. The costs of OUD care vary by injection behavior profile, with additional costs assigned to those with higher-frequency use. There are initial and weekly costs of MOUD and age- and sex-stratified costs of health care services that are not attributable to opioid use or sequelae.

### Model Data

#### Simulated Cohorts

Table 1 outlines key input parameters for the base case and comparison strategies.^27,51,54,55,56,57,58,59,60,61,62,63,64,65,66,67,68,69^ We initialized the model with a cohort of individuals reflecting the age and gender of the US population who inject opioids (informed by the US Census and literature).^28,29,30,31,32,33,34^ We chose the largest cohort size of 2.25 million to produce stable model runs. At model initialization, the mean (range) age of the cohort was 44 (18-99) years, 70% were male, 50% had high-frequency injection use, 10% had low-frequency injection use, and 40% had no current injection use.^28,29,30,31,32,33,34^ We assumed imperfect access to harm reduction services so that 66% of the cohort practiced unsterile injection technique and 35% regularly shared injecting equipment.^70^

**Table 1.** Estimates for Key Model Parameters to Characterize Outcomes of People Who Inject Drugs During a Lifetime^a^

Parameter	Estimate (range)	Source
Population		
Probability of ever drug use	100% of cohort ever injected, age and gender mix informed by literature	Lansky A et al,^28^ 2014; Martin SS et al,^29^ 2017; Degenhardt L et al,^30^ 2017; CDC,^31^ 2018; US Census Bureau,^32^ 2016
Probability of injection drug use frequency	Varies by age and sex	Tan S et al,^33^ 2018; Buresh M et al,^34^ 2019
Needle-sharing prevalence	0.35 (0.18-0.70)	Smith BD et al,^35^ 2013; Abara WE et al,^36^ 2019; Kim NJ et al,^37^ 2015; Asher AK et al,^38^ 2019; Ropelewski LR et al,^39^ 2011
Sequelae of drug use		
Probability of overdose		MDPH,^40^ 2016; MDPH,^41^ 2019; CDC,^42^ 2015; Cedarbaum ER and Banta-Green CJ,^43^ 2016; Hudgins R et al,^44^ 1995; Hser YI et al,^45^ 2017
Low-frequency drug use	0.0026 (0.0026-0.0027)	Hedegaard H et al,^46^ 2018
High-frequency drug use	0.0005 (0.0005-0.0006)	
Probability of fatal overdose	0.1100 (0.1000-0.2200)	Hedegaard H et al,^46^ 2018; MDPH,^47^ 2017; MDPH,^48^ 2019
Proportion of infections attributable to IE	100% (NA)	Assumed
Probability of hospitalization		
After NFOD	0.9700	Unpublished data
For IE	0.1500 (0.1330-0.1670)	N’Guyen Y et al,^23^ 2017
For SSTI	0.0019 (0.0008-0.0040)	Hope VD et al,^22^ 2015
Previous overdose multiplier for risk of subsequent overdose by number of NFODs		
1	1.15 (0.72-1.82)	Caudarella A et al,^49^ 2016
2-3	1.81 (1.19-2.27)	
4-7	2.12 (1.11-4.04)	
≥8	5.24 (1.56-17.01)	
Previous infection multiplier for risk of subsequent infection	2.00 (1.50-5.10)	Alagna L et al,^50^ 2014
Inpatient		
Duration of hospitalization for SSTI, mean, wk	2 (1-4)	Miller AC and Polgreen PM,^51^ 2019
Duration of hospitalization for IE, mean (range), wk	6 (4-8)	Miller AC and Polgreen PM,^51^ 2019
Probability of PDD	0.0300 (0.0125-0.0600)	Meisner JA et al,^52^ 2020; Kimmel SD et al,^53^ 2021
Outpatient addiction care linkages		
Linkage to outpatient addiction care after hospitalization if taking MOUD	0.99 (0.50-0.99)	Expert opinion
Linkage to outpatient addiction care		
Status quo	0.00357 (0.00146-0.01236)	Hall N et al,^54^ 2021; Lewer et al,^55^ 2020
BUP	0.01700 (0.00850-0.03400)	Miller AC and Polgreen PM,^51^ 2019; Lewer et al,^55^ 2020
BUP plus HR	0.01700 (0.00850-0.03400)	Miller AC and Polgreen PM,^51^ 2019; Lewer et al,^55^ 2020
Outpatient MOUD treatment initiation		
Outpatient addiction care	0.30 (0.15-0.60)	Simon CB et al,^27^ 2017
Outpatient unlinking		
Spontaneous unlinkage from outpatient addiction care with MOUD	0.037104 (0.018552-0.074208)	Sohler NL et al,^56^ 2010
Spontaneous unlinkage from outpatient addiction without MOUD	0.123952 (0.061976-0.247942)	Sohler NL et al,^56^ 2010
Mortality		
Background mortality without overdoses	Varies by age and sex (0.0008-0.0011)	Arias E,^57^ 2012; Chang KC et al,^58^ 2017
Probability of death, untreated		
IE	0.1623 (0.0848-0.5358)	Verhagen DW et al,^59^ 2006; Veldhuizen S and Callaghan RC,^60^ 2014
SSTI	0.0023 (0.0012-0.0028)	Veldhuizen S and Callaghan RC,^60^ 2014
Probability of death, inpatient		
With IE	0.0100 (0.0018-0.0161)	Veldhuizen S and Callaghan RC,^60^ 2014; Rodger L et a,^61^ 2018; Cresti A et al,^62^ 2017; Hill EE et al,^63^ 2007; Ternhag A, et al^64^ 2013
With SSTI	0.0006 (0.0004-0.0012)	Veldhuizen S and Callaghan RC,^60^ 2014
With overdose	0.0190 (0.0130-0.0270)	Jiang Y et al,^65^ 2017
Costs, $		
Background	Varies by age and sex	AHRQ^66^
Fatal overdose	460.59 (230.30-690.00)	Behrends CN et al,^67^ 2019
NFOD, not hospitalized	1197 (599-1798)	Behrends CN et al,^67^ 2019
Hospitalization for IE	23 091 (8736-34 410)	Miller AC and Polgreen PM,^51^ 2019
Hospitalization for SSTI	19 001 (9124-26 378)	Miller AC and Polgreen PM,^51^ 2019
Hospitalization for overdose	15 194 (12 744-15 646)	Behrends CN et al,^67^ 2019
Inpatient MOUD	43.63 (21.82-87.26)	US Department of Veterans Affairs^68^
Outpatient addiction visit with MOUD		
Status quo	126.89 (63.44-253.78)	CMS^69^ and expert opinion^b^
BUP	64.64 (32.32-129.28)	CMS^69^ and expert opinion^b^
BUP plus HR	68.58 (34.29-137.16)	CMS^69^ and expert opinion^b^

Abbreviations: AHRQ, Agency for Healthcare Research and Quality; BUP, onsite buprenorphine prescribing; BUP plus HR, onsite buprenorphine plus harm reduction; CDC, Centers for Disease Control and Prevention; CMS, Centers for Medicare & Medicaid Services; IE, infective endocarditis; MDPH, Massachusetts Department of Public Health; MOUD, medication for opioid use disorder; NA, not applicable; NFOD, nonfatal overdose; PDD, patient-directed discharge; SSTI, severe skin and soft tissue infection.

^a^ The the Reducing Infections Related to Drug Use Cost-Effectiveness (REDUCE) model runs on a weekly time cycle; therefore, all probabilities in this table are weekly probabilities. Calibrated inputs have been adjusted to meet the 5 calibration points. See eMethods in Supplement 1 for a more detailed explanation of the calibration targets.

^b^ Consensus obtained among the study authors.

#### Sequelae and Hospitalization

We estimated the rates of fatal and nonfatal overdose from state-level data.^46,47,48^ We used published literature to estimate the rates of injection-related infections and the proportion of infections that are IE and severe SSTIs.^51,71,72,73^

#### Outpatient Services

We used published data to estimate linkage to a PCP, which did not vary by strategy.^74^ For the status quo strategy, we assumed that all patients with OUD would be referred to an offsite addiction specialist for buprenorphine. Each referred individual had a 21% weekly probability of presenting for care,^54^ and those who presented to addiction care had a 30% probability of accepting buprenorphine.^27^ For the BUP and BUP plus HR strategies, we assumed 100% of PCPs would offer buprenorphine at their visits, but patients had a 30% probability of accepting it. We estimated that individuals treated with buprenorphine would be 4 times less likely than those not receiving buprenorphine to unlink from care.^75^ Treatment retention did not differ among the strategies.

#### Mortality

We used overdose-removed US age- and sex-adjusted mortality from the National Vital Statistics System^57^ to derive the competing causes of death parameter. Aside from sequelae, persons who inject opioids could encounter additional drug-related mortality risk (eg, other infections and violence).^76^ We accounted for additional opioid-related harms not captured by fatal OD or sequelae by multiplying the resulting mortality rates by 1.2.^58^

#### Modified Health Care Sector Costs

We derived estimated health insurance costs from the 2021 Laboratory and Physician Fee Schedules from the Centers for Medicare & Medicaid Services^69^ and the Medical Expenditure Panel Survey.^77^ We did not include out-of-pocket costs, hence the labeling as modified health care sector costs. For the 5-year budgetary impact analysis, we estimated costs to PCPs in their personal practice and assumed the costs of a one-time buprenorphine training, the opportunity cost of attending the training, a 1-month reduction in daily patient visits while the practitioner became familiar with buprenorphine prescribing, and direct purchasing costs for harm reduction supplies and naloxone for 30 patients for 5 years.

#### Sensitivity Analyses

We performed deterministic sensitivity analyses (DSAs) to determine the effect of varying model parameters and critical model assumptions. Because there is uncertainty regarding the empirical data used, we performed probabilistic sensitivity analyses (PSAs) to generate quantitative estimates of uncertainty in selected simulated outcomes. For each PSA, we performed 1000 simulations on 1000 people to get a cohort of 1 million persons to ensure a comprehensive range for the parameters that had more uncertainty because of lack of data in the existing literature. We generated a 95% credible interval (Crl) using PSAs for pertinent outcomes. From these PSAs, we also calculated the net monetary benefit of BUP plus HR compared with BUP assuming a willingness-to-pay threshold of $100 000 per LY.

## Results

Using a microsimulation model of 2 250 000 million people in the US who inject opioids (mean [SD] age, 44 [18-99] years; 69% male and 31% female), we found health and cost outcome differences in the 3 modeled strategies. Status quo resulted in 1162 overdose deaths per 10 000 (95% Crl, 1144-2303) people, whereas both BUP and BUP plus HR resulted in approximately 160 fewer deaths per 10 000 people (95% CrI for BUP, 802-1718; 95% CrI for BUP plus HR, 692-1810). In the status quo strategy, mortality rates were 5.76% for SSTIs and 38.94% for IE, both of which were greater than in the BUP (2.10% for SSTIs and 36.34% for IE) and BUP plus HR (2.11% for SSTIs and 36.46% for IE) strategies (Table 2). Compared with the status quo strategy, life expectancy was extended in BUP by 2.65 years and BUP plus HR by 2.71 years (Table 2). Integrating buprenorphine alone extended discounted life expectancy by 0.16 years and buprenorphine combined with harm reduction kits extended discounted life expectancy by 0.17 years. Status quo resulted in 10 957 hospitalizations per 10 000 individuals throughout the lifetime (95% Crl, 14 270-28 839) (Table 3). Because BUP and BUP plus HR extended life, they created more person-time exposed to the risks of drug use and, therefore, paradoxically resulted in more hospitalizations: 1454 more in BUP and 1169 more in BUP plus HR. The BUP strategy resulted in 96 more cases of severe SSTIs per 10 000 individuals than the status quo strategy. In contrast, BUP plus HR resulted in 164 fewer SSTIs per 10 000 people. Both the BUP and BUP plus HR strategies decreased mortality from all sequelae by approximately 33% compared with the status quo strategy.

**Table 2.** Selected Cost and Clinical Outcomes From Base Case Analysis^a^

Scenario	Deaths, No.		Deaths, %			Life expectancy, y	Undiscounted lifetime medical costs per person, $			ICER^b^
	Total	Averted	Overdose	SSTI	IE			Discounted cost per person (95% CrI), $	Discounted LYs (95% CrI)	
Status quo	772 722	NA	11.17	5.76	38.94	70.72	773 000	203 500 (203 000-222 000)	6.56 (6.33-6.74)	NA
BUP	517 795	254 927	11.18	2.10	36.34	73.37	854 900	209 400 (201 000-217 000)	6.73 (6.37-6.77)	Dominated^c^
BUP plus HR	511 900	260 822	11.18	2.11	36.46	73.43	852 000	209 400 (200 300-212 000)	6.73 (6.43-6.76)	34 400

Abbreviations: BUP, onsite buprenorphine prescribing; BUP plus HR, onsite buprenorphine plus harm reduction; CrI, credible interval; ICER, incremental cost-effectiveness ratio; IE, infective endocarditis; LY, life years; NA, not applicable; SSTI, severe skin and soft tissue infection.

^a^ All costs were rounded to the nearest tenths for representation on this table; however, cost and ICER calculations used values to the hundredth decimal point. As such, the ICER for a given alternative is higher than that of the next, more effective alternative.

^b^ The overall ICER was calculated as the difference in the average discounted costs for the total US population divided by the difference in the discounted life expectancy for the total US population, all discounted at 3% per year.

^c^ Costs more and had worse clinical outcomes.

**Table 3.** Selected Clinical Outcomes and Health Care Use Costs From Base Case Analysis

Scenario	Averted mortality per 10 000 people, %			Completed therapy, %		Total costs per per person (95% CrI), $^a^			
	OD	SSTI	IE	SSTI	IE	Hospitalizations	Outpatient	Health care
Status quo	NA	NA	NA	78.8	61.0	67 192 (64 344-142 003)	43 372 (45 537-54 579)	100 564 (109 881-196 582)
BUP	160.9	395.5	576.5	91.3	63.7	65 440 (50 832-101 161)	104 649 (89 055-103 998)	170 089 (139 887-205 159)
BUP plus HR	159.8	399.4	599.4	91.3	63.6	63 860 (49 293-100 293)	111 373 (97 552-113 826)	175 233 (146 845-214 119)

Abbreviations: BUP, onsite buprenorphine prescribing; BUP plus HR, onsite buprenorphine plus harm reduction; CrI, credible interval; IE, infective endocarditis; NA, not applicable; SSTI, severe skin and soft tissue infection.

^a^ The 95% CrIs were derived from the probabilistic sensitivity analysis. The CrIs were not calculated for certain outcomes because they are not primary outcomes from the model but rather were calculated by combining multiple outcomes.

In terms of costs, compared with status quo, the mean undiscounted lifetime medical cost per person was greater for the other strategies: $854 900 for BUP and $852 000 for BUP plus HR (Table 2). The combined total health care costs per person during a lifetime increased with each strategy compared with status quo by 69.1% for BUP and 74.3% for BUP plus HR. We also noted that there was a shift in the cost components away from costs of hospitalization for sequelae of drug use (decreasing per persons by $1752 for BUP and $3332 for BUP plus HR) and toward outpatient costs (increasing by $61 277 per persons for BUP and $68 001 per 10 000 persons for BUP plus HR) (Table 3). Compared with status quo, BUP plus HR had an ICER of $34 400 per LY and was considered the cost-effective strategy; the BUP strategy was more expensive and less effective than the next strategy (Table 2).

We performed 1-way DSAs on parameters that likely affected life expectancy and costs when comparing BUP plus HR to status quo (eTables 1-43 and eFigures 1 and 2 in Supplement 1). In the DSA that assumed lower linkage probability to outpatient addiction care in inpatient or background settings, BUP had the greater cost, more LYs gained, and an ICER of $66 040 when compared with BUP plus HR. The BUP plus HR strategy remained the preferred strategy, with an ICER of $35 570 per LY gained compared with BUP (eTable 18 in Supplement 1). In the DSA assuming lower IE hospitalization rates, BUP was the preferred strategy, with an ICER of $43 244 when compared with status quo (eTable 26 in Supplement 1).

Finally, our PSA showed that under different assumptions about parameter values, some of our clinical outcomes had wide ranges and overlapping CrIs between status quo and other strategies (eTable 45 in Supplement 1). In our PSAs, BUP plus HR was the preferred strategy 76% of the time at a willingness-to-pay threshold of $100 000 per LY. In the budgetary impact analysis from the PCP perspective, we estimated that the total cost of BUP plus HR during a 5-year period to an individual PCP practice was approximately $13 000 (Table 4).^78,79^

**Table 4.** Five-Year Budgetary Impact Analysis From PCP Perspective to Offer Onsite BUP Plus HR

Variable	Estimated cost amount, $	Source
Attending 1-time 8-hour buprenorphine waiver training	149.00	SAMHSA^78^
Opportunity cost to attend an 8-hour buprenorphine training	707.20	SAMHSA^79^
1-Month reduction in volume of daily patient visits to account for providers becoming familiar with buprenorphine prescribing	5000.00	Expert opinion^a^
Direct PCP purchasing of harm reduction supplies and naloxone for a panel of 30 patients	7092.00	Expert opinion^b^

Abbreviations: BUP plus HR, onsite buprenorphine plus harm reduction; PCP, primary care practitioner; SAMHSA, Substance Abuse and Mental Health Services Administration.

^a^ Todd Kerensky, MD, South Shore Health, South Weymouth, Massachusetts, written communication, February 28, 2022.

^b^ S.K., written communication, January 24, 2022.

## Discussion

In this microsimulation modeling study of US people who inject drugs, we estimated the long-term clinical outcomes, costs, and cost-effectiveness of integrating onsite addiction care into primary care. We found that BUP plus HR improved clinical outcomes, averted sequelae, and increased life expectancy compared with status quo and was cost-effective. Our results suggest that integrated addiction care in primary care has the potential to save lives and increase nonemergency health care use, which is consistent with prior literature.^80,81^ Colocated addiction services within primary care is pragmatic and effective and has comparable quality to specialty care.^82^ We found that onsite BUP plus HR provides better outcomes than BUP alone at a lower cost.

Even though treatment with buprenorphine has lifesaving properties on its own, people who are actively using or injecting drugs may not desire pharmacotherapy because of concerns about precipitated withdrawal, practitioner stigma,^10^ lack of health insurance,^83^ and high copayments.^84,85^ Onsite harm reduction tools are not currently part of the traditional medical model^86^; however, integrating harm reduction kits into care could provide patients with treatment autonomy and serve as tools for treatment engagement in times of active and/or chaotic use.^87^ Our model for onsite harm reduction kits provided a fixed quantity of safer injection and wound care equipment that may not last 1 week (or even 1 day) for higher-frequency users. That there was still a mortality benefit is notable. Furthermore, although our study modeled impact of harm reduction kits during office PCP visits, kits could be delivered in other ways, including with nurses (during billable visits) or via outreach staff, which could increase patient engagement in health care but may alter our cost and care use estimates. Future work should also evaluate the impact of BUP plus HR for PCPs in rural areas, which may have limited access to community-based harm reduction organizations and sterile equipment.

Prior studies demonstrated that buprenorphine in outpatient settings is cost-effective.^88,89^ Our findings demonstrate that BUP plus HR is cost-effective from a health care sector perspective, but individual PCPs may shoulder the burden of costs. In our budget impact analysis, we estimated that the 5-year cost to a PCP might reach $13 000, which includes both direct resource and opportunity costs. These costs could be offset by the savings incurred by the health care system. These costs included those for X-waiver training, which has been eliminated; thus, we expect this to cost less. Put another way, our findings inform ways to reinvest health care dollars as financial incentives for PCPs to adopt this new paradigm. Public health departments could provide grants or harm reduction kit supplies directly to PCPs to offset these costs as they do in some places with syringe service programs and/or increase Medicaid reimbursements for providing addiction care in primary care.^90^

Although we found that integrating buprenorphine prescribing and harm reduction kits in primary care will decrease patient mortality from overdose, severe SSTIs, and IE, it paradoxically increases the total number of hospitalizations and overall costs. If more individuals stay alive longer, they have more time to engage in health care during their lives. A single-center study alluded to a similar paradoxical cost benefit with a reduction in hospitalization cost but higher cost for buprenorphine prescriptions at 1 year for integrated buprenorphine in primary care.^91^ Because our study models people throughout a lifetime, the BUP plus HR strategy could save lives, prevent injection-related infections, and offload costs from short-term hospitalizations to outpatient settings. Practically, this also means that people who use drugs could have greater opportunities to engage with outpatient PCPs from whom they could receive care for other chronic illnesses^92^ and receive expedited management of injection-related sequelae, leading to higher infection cure rates. Furthermore, the implications of these averted lengthy hospital stays could financially benefit hospital systems, which may have greater capacity to care for individuals with other conditions.

### Limitations

Our study has several limitations. First, we relied on a single published study^75^ to inform parameters on linkage and retention on buprenorphine therapy. Authors with clinical expertise (R.J., S.L.C., L.R.M., S.D.K., and J.A.B.) note, however, that their clinical practices were within the parameter range, lending validity to the input. Second, although important model parameters were informed by studies of the target population, unmeasured confounders may have impacted the results of these studies. Third, given the heterogeneity of how addiction care is provided in the US, we assumed that in the status quo strategy, all patients access addiction specialty care via a referral mechanism and for simplicity’s sake included buprenorphine as the only MOUD modality. These assumptions could have overestimated the impact in the model. Fourth, for the status quo and BUP strategies, we assumed that individuals have linkage to harm reduction supplies in the community, which did not account for any addiction specialists who may have incorporated harm reduction supplies within their own practice. Despite these limitations, our findings did not qualitatively change in sensitivity analyses when varying assumptions were used. Fifth, it is unclear whether PCPs and/or health care entities will be willing to adopt this intervention, because it may increase workload. The extent to which this intervention is adopted will alter the associated health impact.

## Conclusions

The findings of this decision analytical model suggest that integrating buprenorphine and harm reduction kits in primary care will improve clinical outcomes and modestly increase costs. There is a clinical and cost benefit of adding harm reduction services onsite along with buprenorphine. Providing these lifesaving tools in primary care should be a health care system priority.

## Notes

Supplement 1. **eMethods.** Supplemental Methods **eTable 1.** Calibration Targets **eTable 2.** Cost-effectiveness of Integrated Addiction Services Into Primary Care Assuming Lower Probability of PDD Discharge Rate **eTable 3.** Cost-effectiveness of Integrated Addiction Services Into Primary Care Assuming Higher Probability of PDD Discharge Rate **eTable 4.** Cost-effectiveness of Integrated Addiction Services Into Primary Care Assuming a Lower Probability of Spontaneous “Unlinkage” From Outpatient Addiction Without MOUD **eTable 5.** Cost-effectiveness of Integrated Addiction Services Into Primary Care Assuming Higher Probability of Spontaneous “Unlinkage” From Outpatient Addiction Without MOUD **eTable 6.** Cost-effectiveness of Integrated Addiction Services Into Primary Care Assuming a Lower Probability of Hospitalization for Overdose **eTable 7.** Cost-effectiveness of Integrated Addiction Services Into Primary Care Assuming a Higher Probability of Hospitalization for Overdose **eTable 8.** Cost-effectiveness of Integrated Addiction Services Into Primary Care Assuming a Lower Probability of Inpatient Linkage **eTable 9.** Cost-effectiveness of Integrated Addiction Services Into Primary Care Assuming a Higher Probability of Inpatient Linkage **eTable 10.** Cost-effectiveness of Integrated Addiction Services Into Primary Care Assuming a Lower Probability of Fatal Overdose **eTable 11.** Cost-effectiveness of Integrated Addiction Services Into Primary Care Assuming a Higher Probability of Fatal Overdose **eTable 12.** Cost-effectiveness of Integrated Addiction Services Into Primary Care Assuming a Lower Probability Spontaneous “Unlinkage” From Outpatient Addiction With MOUD **eTable 13.** Cost-effectiveness of Integrated Addiction Services Into Primary Care Assuming a Higher Probability of Spontaneous “Unlinkage” From Outpatient Addiction With MOUD **eTable 14.** Cost-effectiveness of Integrated Addiction Services Into Primary Care Assuming a Lower Inpatient MOUD Cost **eTable 15.** Cost-effectiveness of Integrated Addiction Services Into Primary Care Assuming a Higher Inpatient MOUD Cost **eTable 16.** Cost-effectiveness of Integrated Addiction Services Into Primary Care Assuming a Lower Previous Infection Multiplier for Risk of Subsequent Infection **eTable 17.** Cost-effectiveness of Integrated Addiction Services Into Primary Care Assuming a Higher Previous Infection Multiplier for Risk of Subsequent Infection **eTable 18.** Cost-effectiveness of Integrated Addiction Services Into Primary Care Assuming a Lower Probability of Background or Inpatient Linkage of Outpatient Addiction Care **eTable 19.** Cost-effectiveness of Integrated Addiction Services Into Primary Care Assuming a Higher Probability of Background or Inpatient Linkage of Outpatient Addiction Care **eTable 20.** Cost-effectiveness of Integrated Addiction Services Into Primary Care Assuming Lower Probability of Needle Sharing **eTable 21.** Cost-effectiveness of Integrated Addiction Services Into Primary Care Assuming a Higher Probability of Needle Sharing **eTable 22.** Cost-effectiveness of Integrated Addiction Services Into Primary Care Assuming a Lower Probability of Death, Inpatient With SSTI **eTable 23.** Cost-effectiveness of Integrated Addiction Services Into Primary Care Assuming a Higher Probability of Death, Inpatient With SSTI **eTable 24.** Cost-effectiveness of Integrated Addiction Services Into Primary Care Assuming a Lower Probability of Hospitalization For SSTI **eTable 25.** Cost-effectiveness of Integrated Addiction Services Into Primary Care Assuming a Higher Probability of Hospitalization for SSTI **eTable 26.** Cost-effectiveness of Integrated Addiction Services Into Primary Care Assuming a Lower Probability of Hospitalization for IE **eTable 27.** Cost-effectiveness of Integrated Addiction Services Into Primary Care Assuming a Higher Probability of Hospitalization for IE **eTable 28.** Cost-effectiveness of Integrated Addiction Services Into Primary Care Assuming Lower Cost of Untreated Non-Fatal Overdose **eTable 29.** Cost-effectiveness of Integrated Addiction Services Into Primary Care Assuming a Higher Cost of Untreated Non-Fatal Overdose **eTable 30.** Cost-effectiveness of Integrated Addiction Services Into Primary Care Assuming a Lower Fatal Overdose Cost **eTable 31.** Cost-effectiveness of Integrated Addiction Services Into Primary Care Assuming Higher Fatal Overdose Cost **eTable 32.** Cost-effectiveness of Integrated Addiction Services Into Primary Care Assuming a Lower Probability of Death From Untreated SSTI **eTable 33.** Cost-effectiveness of Integrated Addiction Services Into Primary Care Assuming a Higher Probability of Death From Untreated SSTI **eTable 34.** Cost-effectiveness of Integrated Addiction Services Into Primary Care Assuming a Lower Probability of Death From Untreated IE **eTable 35.** Cost-effectiveness of Integrated Addiction Services Into Primary Care Assuming a Higher Probability of Death From Untreated IE **eTable 36.** Cost-effectiveness of Integrated Addiction Services Into Primary Care Assuming a Lower Cost of Outpatient Addiction Visit With MOUD **eTable 37.** Cost-effectiveness of Integrated Addiction Services Into Primary Care Assuming Higher Cost of Outpatient Addiction Visit With MOUD **eTable 38.** Cost-effectiveness of Integrated Addiction Services Into Primary Care Assuming a Lower Probability of Outpatient Addiction Care From Inpatient Care (No MOUD) Without ACS **eTable 39.** Cost-effectiveness of Integrated Addiction Services Into Primary Care Assuming a Higher Probability of Outpatient Addiction Care From Inpatient Care (No MOUD) Without ACS **eTable 40.** Cost-effectiveness of Integrated Addiction Services Into Primary Care Assuming a Lower Probability of Linkage to Outpatient Addiction Care (MOUD) From Inpatient Without ACS, With MOUD **eTable 41.** Cost-effectiveness of Integrated Addiction Services Into Primary Care Assuming a Higher Probability of Linkage to Outpatient Addiction Care (MOUD) From Inpatient Without ACS, With MOUD **eTable 42.** Model Parameters, Data Type Used to Inform Parameters, and Sampling Distributions for Probabilistic Sensitivity Analyses **eTable 43.** Model Outcomes and Credible Intervals for Status Quo, BUP, and BUP + HR **eFigure 1.** Lifetime Clinical Outcomes Cases Averted per 10,000 in BUP, BUP + HR Compared to Status Quo Strategies **eFigure 2.** Deterministic Sensitivity Analysis- Tornado Chart of BUP + HR versus SQ

Supplement 2.
Data Sharing Statement
