# Error in Table 3

**DOI:** 10.1001/jamanetworkopen.2023.15795

**Published:** 2023-05-11

In the Original Investigation titled “Estimated Clinical Outcomes and Cost-effectiveness Associated With Provision of Addiction Treatment in US Primary Care Clinics,”^1^ published April 12, 2023, in Table 3, a percentage symbol had been incorrectly printed in the column heading “Averted mortality per 10 000 people.” This article has been corrected.^1^
