# Supplementary material for: Estimated Clinical Outcomes and Cost-effectiveness Associated With Provision of Addiction Treatment in US Primary Care Clinics
Source: JAMA Netw Open. 2023 Apr 12;6(4):e237888. doi: 10.1001/jamanetworkopen.2023.7888 (PMC10098970; doi:10.1001/jamanetworkopen.2023.7888)
Supplement: Supplement 1. — eMethods. Supplemental Methods eTable 1. Calibration Targets eTable 2. Cost-effectiveness of Integrated Addiction Services Into Primary Care Assuming Lower Probability of PDD Discharge Rate eTable 3. Cost-effectiveness of Integrated Addiction Services Into Primary Care Assuming Higher Probability of PDD Discharge Rate eTable 4. Cost-effectiveness of Integrated Addiction Services Into Primary Care Assuming a Lower Probability of Spontaneous “Unlinkage” From Outpatient Addiction Without MOUD eTable 5. Cost-effectiveness of Integrated Addiction Services Into Primary Care Assuming Higher Probability of Spontaneous “Unlinkage” From Outpatient Addiction Without MOUD eTable 6. Cost-effectiveness of Integrated Addiction Services Into Primary Care Assuming a Lower Probability of Hospitalization for Overdose eTable 7. Cost-effectiveness of Integrated Addiction Services Into Primary Care Assuming a Higher Probability of Hospitalization for Overdose eTable 8. Cost-effectiveness of Integrated Addiction Services Into Primary Care Assuming a Lower Probability of Inpatient Linkage eTable 9. Cost-effectiveness of Integrated Addiction Services Into Primary Care Assuming a Higher Probability of Inpatient Linkage eTable 10. Cost-effectiveness of Integrated Addiction Services Into Primary Care Assuming a Lower Probability of Fatal Overdose eTable 11. Cost-effectiveness of Integrated Addiction Services Into Primary Care Assuming a Higher Probability of Fatal Overdose eTable 12. Cost-effectiveness of Integrated Addiction Services Into Primary Care Assuming a Lower Probability Spontaneous “Unlinkage” From Outpatient Addiction With MOUD eTable 13. Cost-effectiveness of Integrated Addiction Services Into Primary Care Assuming a Higher Probability of Spontaneous “Unlinkage” From Outpatient Addiction With MOUD eTable 14. Cost-effectiveness of Integrated Addiction Services Into Primary Care Assuming a Lower Inpatient MOUD Cost eTable 15. Cost-effectiveness of Integrated Addiction Services Into [file jamanetwopen-e237888-s001.pdf]

Supplementary Online Content

Jawa R, Tin Y, Nall S, et al. Estimated clinical outcomes and cost-effectiveness associated with provision of addiction treatment in US primary care clinics. *JAMA Netw Open*. 2023;6(4):e237888. doi:10.1001/jamanetworkopen.2023.7888

eMethods. Supplemental Methods
eTable 1. Calibration Targets
eTable 2. Cost-effectiveness of Integrated Addiction Services Into Primary Care Assuming Lower Probability of PDD Discharge Rate
eTable 3. Cost-effectiveness of Integrated Addiction Services Into Primary Care Assuming Higher Probability of PDD Discharge Rate
eTable 4. Cost-effectiveness of Integrated Addiction Services Into Primary Care Assuming a Lower Probability of Spontaneous "Unlinkage" From Outpatient Addiction Without MOUD
eTable 5. Cost-effectiveness of Integrated Addiction Services Into Primary Care Assuming Higher Probability of Spontaneous "Unlinkage" From Outpatient Addiction Without MOUD
eTable 6. Cost-effectiveness of Integrated Addiction Services Into Primary Care Assuming a Lower Probability of Hospitalization for Overdose
eTable 7. Cost-effectiveness of Integrated Addiction Services Into Primary Care Assuming a Higher Probability of Hospitalization for Overdose
eTable 8. Cost-effectiveness of Integrated Addiction Services Into Primary Care Assuming a Lower Probability of Inpatient Linkage
eTable 9. Cost-effectiveness of Integrated Addiction Services Into Primary Care Assuming a Higher Probability of Inpatient Linkage
eTable 10. Cost-effectiveness of Integrated Addiction Services Into Primary Care Assuming a Lower Probability of Fatal Overdose
eTable 11. Cost-effectiveness of Integrated Addiction Services Into Primary Care Assuming a Higher Probability of Fatal Overdose
eTable 12. Cost-effectiveness of Integrated Addiction Services Into Primary Care Assuming a Lower Probability Spontaneous "Unlinkage" From Outpatient Addiction With MOUD
eTable 13. Cost-effectiveness of Integrated Addiction Services Into Primary Care Assuming a Higher Probability of Spontaneous "Unlinkage" From Outpatient Addiction With MOUD
eTable 14. Cost-effectiveness of Integrated Addiction Services Into Primary Care Assuming a Lower Inpatient MOUD Cost
eTable 15. Cost-effectiveness of Integrated Addiction Services Into Primary Care Assuming a Higher Inpatient MOUD Cost
eTable 16. Cost-effectiveness of Integrated Addiction Services Into Primary Care Assuming a Lower Previous Infection Multiplier for Risk of Subsequent Infection
eTable 17. Cost-effectiveness of Integrated Addiction Services Into Primary Care Assuming a Higher Previous Infection Multiplier for Risk of Subsequent Infection
eTable 18. Cost-effectiveness of Integrated Addiction Services Into Primary Care Assuming a Lower Probability of Background or Inpatient Linkage of Outpatient Addiction Care
eTable 19. Cost-effectiveness of Integrated Addiction Services Into Primary Care Assuming a Higher Probability of Background or Inpatient Linkage of Outpatient Addiction Care
eTable 20. Cost-effectiveness of Integrated Addiction Services Into Primary Care Assuming Lower Probability of Needle Sharing
eTable 21. Cost-effectiveness of Integrated Addiction Services Into Primary Care Assuming a Higher Probability of Needle Sharing
eTable 22. Cost-effectiveness of Integrated Addiction Services Into Primary Care Assuming a Lower Probability of Death, Inpatient With SSTI
eTable 23. Cost-effectiveness of Integrated Addiction Services Into Primary Care Assuming a Higher Probability of Death, Inpatient With SSTI
eTable 24. Cost-effectiveness of Integrated Addiction Services Into Primary Care Assuming a Lower Probability of Hospitalization For SSTI

© 2023 Jawa R et al. *JAMA Network Open.*

eTable 25. Cost-effectiveness of Integrated Addiction Services Into Primary Care Assuming a Higher Probability of Hospitalization for SSTI
eTable 26. Cost-effectiveness of Integrated Addiction Services Into Primary Care Assuming a Lower Probability of Hospitalization for IE
eTable 27. Cost-effectiveness of Integrated Addiction Services Into Primary Care Assuming a Higher Probability of Hospitalization for IE
eTable 28. Cost-effectiveness of Integrated Addiction Services Into Primary Care Assuming Lower Cost of Untreated Non-Fatal Overdose
eTable 29. Cost-effectiveness of Integrated Addiction Services Into Primary Care Assuming a Higher Cost of Untreated Non-Fatal Overdose
eTable 30. Cost-effectiveness of Integrated Addiction Services Into Primary Care Assuming a Lower Fatal Overdose Cost
eTable 31. Cost-effectiveness of Integrated Addiction Services Into Primary Care Assuming Higher Fatal Overdose Cost
eTable 32. Cost-effectiveness of Integrated Addiction Services Into Primary Care Assuming a Lower Probability of Death From Untreated SSTI
eTable 33. Cost-effectiveness of Integrated Addiction Services Into Primary Care Assuming a Higher Probability of Death From Untreated SSTI
eTable 34. Cost-effectiveness of Integrated Addiction Services Into Primary Care Assuming a Lower Probability of Death From Untreated IE
eTable 35. Cost-effectiveness of Integrated Addiction Services Into Primary Care Assuming a Higher Probability of Death From Untreated IE
eTable 36. Cost-effectiveness of Integrated Addiction Services Into Primary Care Assuming a Lower Cost of Outpatient Addiction Visit With MOUD
eTable 37. Cost-effectiveness of Integrated Addiction Services Into Primary Care Assuming Higher Cost of Outpatient Addiction Visit With MOUD
eTable 38. Cost-effectiveness of Integrated Addiction Services Into Primary Care Assuming a Lower Probability of Outpatient Addiction Care From Inpatient Care (No MOUD) Without ACS
eTable 39. Cost-effectiveness of Integrated Addiction Services Into Primary Care Assuming a Higher Probability of Outpatient Addiction Care From Inpatient Care (No MOUD) Without ACS
eTable 40. Cost-effectiveness of Integrated Addiction Services Into Primary Care Assuming a Lower Probability of Linkage to Outpatient Addiction Care (MOUD) From Inpatient Without ACS, With MOUD
eTable 41. Cost-effectiveness of Integrated Addiction Services Into Primary Care Assuming a Higher Probability of Linkage to Outpatient Addiction Care (MOUD) From Inpatient Without ACS, With MOUD
eTable 42. Model Parameters, Data Type Used to Inform Parameters, and Sampling Distributions for Probabilistic Sensitivity Analyses
eTable 43. Model Outcomes and Credible Intervals for Status Quo, BUP, and BUP + HR
eFigure 1. Lifetime Clinical Outcomes Cases Averted per 10,000 in BUP, BUP + HR Compared to Status Quo Strategies
eFigure 2. Deterministic Sensitivity Analysis- Tornado Chart of BUP + HR Versus SQ

This supplementary material has been provided by the authors to give readers additional information about their work.

eMethods. Supplemental Methods

Introduction

The analyses reported in the main manuscript use the Reducing Infections Related to Drug Use Cost Effectiveness (REDUCE) Model of acquisition and treatment for bacterial infections and overdose associated with injection drug use. The REDUCE model tracks several clinical outcomes including number of people with infective endocarditis (IE), severe skin and soft tissue infections (SSTI), and overdose (OD) (otherwise known as 'sequelae'), number of cases identified, number linked to inpatient and outpatient care, number of people initiating therapy, and number achieving cure from their sequelae of drug use. The model also tracks sequelae-related mortality, undiscounted life expectancy, discounted lifetime medical costs from the health system perspective, and non-discounted program costs from the payer perspective (for interventions designed to improve follow-up). This technical appendix provides details on key features of the model and modeling approach used for this analysis. We constructed the model and performed analyses using C++ and R (3.2.2). The model is available for review upon discussion with the authors and as resources are available. We did not use every component of the model for the current analysis. In addition, we provide figures and several tables detailing calibration, input parameter values and additional results cited in the manuscript.

REDUCE Model

The REDUCE model is an individual-based, stochastic simulation model of the natural history of injection drug use designed to estimate the outcomes and costs associated with various strategies of prevention, treatment, and improving drug use-related care. The model uses a cycle length of one week. Injection frequency is usually reported as injection frequency in the past month" and while frequency may change daily depending on drug availability, most frequency use is somewhat stable over a one-week period.

Overview. The model is designed as a number of modules through which simulated individuals pass. Briefly, a cohort module helps to "create" the population of interest. Next, individuals created during cohort generation enter the "sequelae of drug use (SDU)" module, which is where they encounter probabilities of fatal or nonfatal OD, IE, or SSTI. From the SDU module, individuals enter back into the simulation or link to the "inpatient" module. In the "inpatient" module, individuals are hospitalized for their SDU. There are a variety of interventions (beyond standard hospital treatment) that individuals may encounter if those services are turned "on" by the user. Following the inpatient module, individuals have a probability of linking to outpatient care in the "outpatient" module. Linkage to outpatient care may vary based on the type of services an individual encountered in the hospital and/or the type of SDU they have (overdose vs infection). They may unlink from the outpatient module or never enter it (based on probabilities). The "behavioral transitions" module is when individuals have the probability of moving between injection frequency drug use states (high frequency, low frequency, or no current drug use), between sterile injection practice states (skin cleaning or no skin cleaning), and sharing/reusing needles. After the "behavioral transitions" module, individuals move to the "mortality, cost, and quality of life" module. At this point, the model begins again in cycle n+1.

Module 1: Cohort initiation. When the model is initiated, a cohort of individuals is generated using 6 parameters:

(1) ever injection drug use status (ever/never)
(2) age (18-99)
(3) sex (male/female)
(4) injection frequency (high/low/no current/never)

© 2023 Jawa R et al. *JAMA Network Open.*

(5) reusing/sharing equipment (yes/no/never)
(6) sterile injection practice (cleaning/no cleaning/never)

From these parameters, the cohort developed is a distribution of people who have ever or never injected drugs and those who are ever are stratified by injection frequency and injection practices. The model is structured such that first the user specifies the proportion of the population that has ever injected drugs. Following that, there are two methods by which the model can draw age and sex. The first is by using age/sex tables and the second is by directly specifying age and sex distribution parameters. In the latter method of drawing from age and sex, the user inputs values directly into the deterministic parameter file. These inputs include proportion male, average male age, standard deviation male age, average female age, standard deviation female age, and minimum age

Next, among those who are ever drug users, the probability of injection frequency is drawn from an age/sex stratified table—high, low, and no current injection drug use. For this, all three probabilities of an age/sex group should equal to 1 and the model draws from this set of probabilities. The model does not allow for the added probability to be greater than 1. Finally, all persons who are ever drug users, are assigned an initial status of being a skin cleaner and a needle sharer which does not depend on age and gender. While these are the initial attributes, all individuals have the possibility of "picking up" additional attributes as they move through the model. All never drug users are assigned "never" injection frequency, skin cleaning and needle sharing status.

Assumptions built into the model for the initial cohort:

1) no one starts on treatment for opioid use disorder
2) no one starts out with a history of overdose
3) no one starts with a history of infection
4) no one begins in care or in the hospital setting.

Module 2. Sequelae of Drug Use. Once the cohort is initialized and each individual has been assigned an initial drug use status, age, sex, and injection frequency and practices, individuals enter the SDU module. As stated above, the SDU in this model include IE, SSTI, and OD. When they first enter, the model checks their ever/never status. If they are "never," then they return to the simulation. Therefore, only "ever" drug users can progress through this module. The model then checks their injection frequency. If they are "no current," then they return to the simulation. Therefore, only "low frequency" and "high frequency" injectors progress through this module. Additionally, if the individual is currently in inpatient care, they return to the simulation. If a person is currently on antibiotics, they progress through the SDU module but they cannot acquire a new infection (SSTI or IE).

At this point, remaining individuals are subject to probabilities for acquiring an SDU. On the first cycle of this model, no one has a history of SDU, but have the possibility of acquiring one or multiple through their life. History of SDU is tracked as it has implications for future SDU. One assumption of the model is that in each cycle, an individual can have more than one SDU, but only acquires one infection at a time. Additionally, individuals may have concurrent SDU (meaning that they may acquire IE in cycle 1 and then SSTI in cycle 2, if they have not been hospitalized for their existing SDU. Another assumption of the model is that SDUs can only be acquired while not "inpatient" or on antibiotics (next module).

Individuals who are eligible for an SDU, progress through a number of probabilities of acquiring an SDU. All SDUs are stratified by injection frequency (high and low) and the infectious SDU are also stratified by injection practices (skin cleaning, needle sharing). SDU probabilities are not stratified by age and sex. The model is structured such that an individual first encounters a

© 2023 Jawa R et al. *JAMA Network Open.*

combined probability of OD (fatal + nonfatal), stratified by injection frequency. If an individual has a current infection their OD rate is multiplied by the current infection multiplier. A proportion of ODs are fatal and a proportion are nonfatal. If a person draws an OD, the model checks whether they are within their OD education naloxone distribution effective cycles and dependent on that, fatal OD is drawn. One aspect of the model is that at this point, if a person draws a fatal OD then they are flagged as "dead, fatal OD." They continue to proceed through the rest of the modules but cannot acquire any further attributes (e.g., they cannot get another infection, be hospitalized, start MOUDs, change their behaviors). These individuals, however, accrue the full costs of the cycle (based on background costs, costs of fatal OD, and costs of any other SDUs that are untreated) and utilities (based on age, sex, and other current health states at the end of the cycle). For those that have a nonfatal OD or do not have an OD, they then face a combined probability of infectious SDU (IE + SSTI), stratified by injection frequency, skin cleaning and needle sharing attributes. A proportion of that probability is IE and the other is SSTI. The model is structured to account for a history of SDU (treated in hospital, resolved because it was a nonfatal OD) and for existing SDUs. An existing SDU is anything that an individual has during the current cycle. From a clinical perspective, this represents an "untreated" infection (e.g., someone has not gone to the hospital for their IE or someone is currently on outpatient antibiotics but not cured) or a current nonfatal overdose. Once treatment is complete or the SDU resolves (as is the case with nonfatal OD which resolves in 1 cycle), then the person is flagged with a history of the corresponding SDU.

An existing SDU causes a change in the likelihood of another SDU. In the model, there is a single multiplier for one or more existing SDUs that is applied to both the probability of OD and the probability of infectious SDU. This multiplier exists until the individual is treated for the SDU. For those who have a nonfatal OD, the existing SDU multiplier will be applied to the probability of infection in the same cycle only since an existing nonfatal OD (that does not link to inpatient), only lasts one cycle. Additionally, a history of SDUs changes the probability of future SDUs. Multipliers are only applied to the SDU for which there is a history (e.g., OD history changes the probability of recurrent OD; any infection history changes the probability of future infection [any infection, not just the one that occurred]). For OD, there are 4 multipliers (e.g., 1 past nonfatal OD, 2-3 past nonfatal OD, 4-7 past nonfatal OD, and 8+ past nonfatal ODs). For history of treated infections, there is only one multiplier (1+ past treated infections). For instance, in cycle 1, an individual gets IE but does not go to the hospital/receive treatment and does not die in cycle 1. By cycle 2, having IE makes that individual have a greater probability of OD or SSTI. For this model, individuals will not be able to acquire the same SDU in that next cycle. From the previous example, the individual with IE will only be able to acquire OD or SSTI, not IE in cycle 2. While that infection remains untreated, there is an effect on getting another infection/OD. Once that infection is treated, then there is a separate effect of this infection on future infections. Therefore, this module has two multipliers: 1) one that can change the probability of an additional SDU if current SDU is untreated, and 2) one that can change the probability of a recurrent SDU (in the future) if the current SDU is fully treated and they survive it.

If an individual does not acquire an SDU in the current cycle and does not have an untreated SDU from a past cycle, they return to the simulation. If they acquire one or more SDUs, or have an untreated SDU from a past cycle, then individuals draw linkage probability to inpatient from the SDU. Linkage to inpatient depends on the linkage probability of their SDU; if an individual has more than one SDU, their linkage probability is the highest of the linkage probabilities for the SDUs they have. There remains the possibility that an individual does not link to inpatient. In the case of nonfatal OD, it implies that the OD was not severe enough to require hospitalization (or was treated in the field). In the subsequent cycle, there should not be a flag for untreated overdose. All nonfatal overdoses are, by definition, treated so the "existing" state can only last for the cycle in which the non-fatal overdose occurs. In the case of endocarditis, the untreated

© 2023 Jawa R et al. *JAMA Network Open.*

flag should remain on until the person either dies or links to inpatient care and gets cured. This is because endocarditis is generally uniformly fatal if untreated. In the case of SSTI, some SSTIs can spontaneously clear (e.g., consider a pimple or slight redness around a cut). In the model, we are assuming that SSTIs being modeled are serious infections that would require hospitalization or, otherwise, ultimately lead to death. Therefore, similar to IE, the untreated SSTI flag remains on until either 1) the person dies, or 2) the person is linked to inpatient and cured, whichever occurs first. Individuals who go to the hospital will be classified as “inpatient” starting in the same cycle and will have an “in-hospital mortality.” Once they leave the hospital, they are considered as having a history of infection. If an individual does not link to inpatient, they are classified as having “existing” SDU and have different risks of death (untreated mortality probabilities for each SDU). Individuals who come to the SDU module on subsequent cycles with an additional SDU (>1 SDU at a time) will have the probability of hospitalization that is equal to the highest probability of the SDUs.

Attributes that an individual can acquire in this module and are tracked:

1. Current IE
2. Current SSTI
3. Current OD, non-fatal
4. Current OD, fatal
5. History of treated IE
6. History of treated SSTI
7. History of treated OD

Module 3. Inpatient Hospitalization Module. One assumption of the model is that any individual that is either a) current injection drug use or b) has a current, untreated SDU is presumed to have opioid use disorder (OUD). Some sequelae of OUD are infectious (SSTI and IE) and some are non-infectious (e.g., OD).

Each individual with 1+ SDU has a probability per cycle of presenting to an inpatient setting for their care. When individuals enter the inpatient module, the model checks their current SDU status. If they do not have a current untreated SDU or died of fatal OD in the previous module, or they are on outpatient antibiotics, then they return to the simulation. Therefore, only those individuals with active SDU can progress through this module.

The path through the inpatient module is conditional on the SDU(s) that an individual has: nonfatal OD, IE, SSTI, or combination. The hospitalization duration for OD is 1 cycle; the hospitalization duration for SSTI and IE are drawn stochastically from a normal distribution with a user defined mean and standard deviation; the model allows for a maximum hospitalization to be set so that at the end of the max amount of time a person will leave the hospital. Each hospitalization is associated with a cost that is accrued in later module. The key feature of this module is that individuals may encounter a variety of in-hospital services. These services are either turned on or off by the user depending on the analysis. If they are on, then individuals will have a probability of being offered and of accepting those services during their hospitalization. Each service has an effect either within this module or elsewhere in the simulation. Each service is associated with a cost that is applied in a separate module at the end of the simulation. Individuals should be “marked” as using/receiving a service such that the cost can be tabulated in the separate module.

Each individual has a probability of in-hospital mortality that is discussed in detail in the mortality module section. It is mentioned here to note that it is an attribute that an individual can acquire. During hospitalization, individuals “carry” a flag/marker that designates them as hospitalized. While hospitalized, individuals cannot get a new SDU so they will not enter SDU module. They

© 2023 Jawa R et al. *JAMA Network Open.*

have an “in hospital” mortality that is conditional on the SDU for which they are hospitalized. For the duration of their hospitalization, their injection frequency is considered to be “no current” regardless of their actual status and they are not exposed to behavior transitions. The exception to this rule is as follows: In the last hospitalization cycle, individuals are exposed to behavior transitions based on their pre-hospitalization status. If they have received any intervention that would affect their behaviors (MOUD, skin cleaning education or clean needle distribution), the intervention effect will be applied to their actual or pre-hospitalization behaviors and post-treatment effective cycles will be drawn. These behavioral changes are assigned in the last inpatient cycle so that they take effect the first cycle out of inpatient. However, cost-life-mortality module still consider them as “no current”. When the inpatient hospitalization time has lapsed, then individuals move to the outpatient module. In the outpatient module, they have a probability of then linking to different types of care.

Module 4. Outpatient Care Module. There are two different ways in which an individual can enter the outpatient module. First, an individual can enter via background linkage. This means that those who are not hospitalized but “decide” to seek care can do so by entering this module. Second, an individual can enter via the inpatient module.

For individuals entering from the simulation (background). Each individual encounters the outpatient module. Individuals with a “death” flag from a previous module (fatal OD) enter the outpatient module and immediately return to the simulation. Individuals who are currently hospitalized immediately return to the simulation. All other “ever” drug user individuals have a probability of linking to outpatient care and progress through the outpatient module, regardless of history of SDU or drug use status. If individuals do not draw “linkage” then they return to the simulation.

For individuals entering from the inpatient module (inpatient linkage). When the inpatient hospitalization time has lapsed, then individuals encounter a linkage probability to the outpatient module depending on the inpatient services they have received.

Outpatient addiction care. Individuals have a probability of linking to outpatient addiction care (either with or without MOUDs). One cannot be simultaneously in outpatient addiction care with MOUDs and without MOUDs (these are separate states). But individuals can be simultaneously in outpatient addiction care (with or without MOUDs). Individuals can link to outpatient addiction care either from their addiction specialty provider or from the primary care provider. If they link to addiction care (irrespective of at PCP office or in addiction specialist office) and aren’t linked to MOUD, they decrease their probability of unclean injections and needle sharing. If they link to MOUD and addiction care (irrespective of at PCP office or in addiction specialist office), there are downstream model effects such as decreased probability of unclean injection; decreases needle sharing, increases the probability of moving to a lower frequency state, and decreases the probability of moving out of no/low frequency state. The descriptions of visit type, frequency, and diagnostic tests offered, and harm reduction supplies offered that we assumed for this model are reflected below.

	**Primary Care**	**Addiction specialty care**
**Intake Visit**	Once per lifetime<br>50% Level 3, 50% Level 4	Once per lifetime<br>50% Level 3, 50% Level 4

© 2023 Jawa R et al. *JAMA Network Open.*

**Routine Visit Frequency**	3 weekly MD visits in<br>1st month following intake<br>Monthly MD visits for following 11 months<br>50% Level 3, 50% Level 4	3 weekly MD visits in 1st month following intake<br>Biweekly MD visits for the following 11 months<br>50% Level 3, 50% Level 4
**Tests (HIV, Hepatitis A/B/C virus, syphilis, Gonorrhea, Chlamydia screening)**	All happen at intake, then once annually	All happen at intake, then once annually
**Urine toxicology screen**	Monthly at MD visits	Monthly at MD visits
**Harm reduction supplies**	Monthly at MD visits	NA
**Intranasal naloxone**	Given once annually at intake	NA

Individuals have a probability of unlinking from outpatient addiction care either with or without MOUDs or transitioning between MOUD states. There is a separate probability of linking to outpatient addiction care (with or without MOUDs) for those coming from the inpatient module and those coming from the simulation (spontaneous linkage/background linkage).

If an individual is in outpatient addiction care and acquires an infection (SSTI or IE) they will automatically be linked to inpatient care in the next cycle. In this case, they will unlink from outpatient care and all outpatient-related flags/cycles will be cleared.

Module 5: Behavioral Transitions Module. Following the inpatient and outpatient modules, individuals move to the behavioral transitions module. Individuals may also enter this module "from the simulation." The latter represents the ability of someone to change their behaviors organically (without interventions). This is the module in which they can move between high frequency, low frequency, and no current use states, move from never and ever IDU, move between skin cleaning and not skin cleaning states, and move between sharing needles and not sharing needles states. There is a prior probability of movement between states (status quo) and various "flags" acquired throughout the model progression that impact certain probabilities. These have been outlined in various other module descriptions but are also be outlined below.

Treatment Effects: The primary driver of morbidity and mortality in the module is the injection frequency. High frequency individuals are at higher risk than low frequency injectors of SDU in this model. All persons who are "ever" injectors have the possibility of moving to a higher or lower injection frequency state (depending on their current state) or staying in their current state per cycle. For instance, a high frequency injector may remain as a high frequency injector or may move to low frequency or no current use states. There are a few ways that the injection frequency can be modified in the model. <u>In brief, however, only acute inpatient hospitalization and linkage to MOUD can change injection frequency in the model.</u>

Mechanisms by which transitions between injection frequency states are changed:

1) Hospitalization.
2) Outpatient MOUD initiation.
3) Behavioral transitions with MOUD.

© 2023 Jawa R et al. *JAMA Network Open.*

Module 6: Mortality Module.

Mortality. There are two places in the model that an individual can die: fatal OD in the SDU module and in the mortality module. To review, in the SDU module, an individual draws a combined probability of all types OD which is stratified by injection frequency (high and low frequency). From that combined probability, an individual can draw either a fatal or non-fatal overdose. If an individual draws a fatal overdose, then go through the remainder of the cycle with a “fatal OD” flag up which does not allow them to get any further interventions, collect additional costs, change their behavior status, etc., however, they will accumulate the background cost and utility of that cycle. As such, the background mortality in the mortality module should exclude OD mortality.

The background mortality risk is an age and sex adjusted mortality probability (excluding fatal OD). There are a number of occurrences in the model that can impact the weekly risk of mortality. First, individuals who are hospitalized for an SDU have an increased risk of death. If the inpatient individual further gets an ID consult, their infection inpatient mortality rate is augmented by an ID consult mortality multiplier (ID consult will not affect OD mortality). Second, individuals who have an untreated SSTI or untreated IE have an increased risk of death. These risks are input as probabilities (and converted to rates by the model) which are then *added* to the background mortality at the end of each cycle. Once a patient is cured of their infection their SDU flags are removed and their mortality goes back to background mortality. The mortality risk only applies for each cycle that they have that risk. For example, a person gets IE and does not present to inpatient care during a cycle. Then they have an “existing IE” flag that the end of the cycle should prompt the rate of death for untreated endocarditis to be added to the background mortality. On cycles 2-5 that same individual, however, is hospitalized and being treated for their IE. For those cycles, they get an “in-hospital for IE” flag such that the in-hospital IE mortality rate is added to their background mortality each cycle. On cycle 6, this person leaves the inpatient setting (completes treatment) so all flags are, therefore, off and at the end of that cycle they get only background mortality. We do not include an additional mortality risk for being an active drug user since most of that risk will be folded into overdose and other SDUs.

Cause of death as an output: In the model, individuals can die of background causes or as a direct result of their injection drug use. Direct causes of injection drug use include:

1. OD (combination of fatal OD/ hospitalized and nonfatal OD that dies in the hospital)
2. IE(combination of hospitalized and non-hospitalized)
3. Severe SSTI (combination of hospitalized and non-hospitalized)

Aside from fatal OD, all of the other causes of death get added to the background mortality as outlined above. For instance, an individual’s weekly probability of death (conditional on not dying of a fatal OD) may be p_d and they may have endocarditis which increases their risk of death by *x.* The individual’s weekly risk of death is, therefore, *the sum of the rates converted to a probability.* However, as an output, we need to be able to determine the attributable cause of death (this person may have died of IE OR background causes). To do this, we use the sum of the rates as the denominator and the individual mortality risk (rates) as the numerator in drawing the cause of death. Important for consistency, the input parameters are probabilities and therefore all rates are calculated in the model. For instance, background mortality rate is x (prob = p_x), untreated IE is y (prob = p_y), and untreated SSTI is z (prob = p_z). Total rate = x + y + z. This is converted to a probability of death, p_c. If death = yes, then to determine the cause, the model draws from the following probabilities: probability that death was from background = r_x/r_c; probability that death was from IE = r_y/r_c; probability that death was from SSTI = r_z/r_c (Cause of

© 2023 Jawa R et al. *JAMA Network Open.*

death should be calculated based on rate ratios) The same methodology would hold true if there were two concurrent SDUs. As outputs, we only want to output background deaths, IE deaths, SSTI deaths, and OD deaths (do not need to stratify by inpatient vs outpatient).

Costs. Costs are accrued for a variety of reasons. At the end of each cycle, costs associated with certain characteristics should be added up to the background costs. A discount rate should be applied at that time.

1. Background costs: age and sex stratified costs of being alive which are the same for never and ever injection drug use
2. Injection drug use costs: Ever injection drug users should have costs that are stratified by frequency:
   a. Cost of no current injection drug use
   b. Cost of high frequency injection drug use
   c. Cost of low frequency injection drug use
3. Cost of fatal OD
4. Cost of non-fatal OD not hospitalized
5. Cost of untreated IE
6. Cost of untreated SSTI
7. Per cycle costs of hospitalization for IE*
8. Per cycle costs of hospitalization for SSTI*
9. Per cycle costs of hospitalization for OD*
10. Inpatient services costs
    a. MOUDs: recurring weekly cost while inpatient
11. Outpatient services costs
    a. Outpatient addiction with MOUD: recurring weekly cost while on MOUD and linked to addiction care
    b. Outpatient addiction without MOUD: recurring weekly cost while linked to care

*If someone is hospitalized for multiple causes (IE, SSTI, OD) they do not get costs for all 3 as we would be double (or triple) counting. Instead, they get the maximum of the hospitalization costs for what they have (i.e., if the individual was hospitalized for SSTI and IE, they would receive whichever costs are higher, SSTI or IE, but not costs for both).

All costs and life expectancy should have a discount rate applied at the end of the cycle so that we can derive a discounted cost and a discounted life expectancy.

© 2023 Jawa R et al. *JAMA Network Open.*

eTable 1. Calibration Targets

Target	Target Value	Modeled Value	Standard Error
**1-year IE incidence among PWID**	55 per 10,000	50 per 10,000	9.1%
**1-year fatal OD incidence among PWID**	68 per 10,000	70 per 10,000	2.9%
**3-year IE mortality among PWID**	29.0%	35.7%	23%
**Remaining life expectancy among PWID**	72.3 years	70.5 years	2.5%
**1-year % remain disengaged from treatment**	89%	88%	1.1%

IE = infective endocarditis, OD= overdose, PWID = People who inject drugs

Comment: There are few data on long-term IE-related incidence and mortality specific to PWID opioids. We calibrated our model to five targets among persons who inject opioids: 1-year endocarditis and fatal overdose incidence, 3-year endocarditis mortality, life expectancy, and remaining disengaged from addiction treatment. We varied parameters within feasible ranges to fit our model to these targets. We then validated our model following best practices for model validation. After assessing the internal validity of the model, we externally validated by comparing our model-generated outcomes to published literature.

© 2023 Jawa R et al. *JAMA Network Open.*

eTable 2. Cost-effectiveness of Integrated Addiction Services Into Primary Care Assuming Lower Probability of PDD Discharge Rate

**Testing Strategy**	Cost, $‡	Incremental Cost, $‡	Incremental Life Years (LY) ‡	ICER ($/LY) ‡
**Status Quo**	$ 203,799	--	--	--
**BUP**	$ 209,697	--	--	Dominated†
**BUP plus HR**	$ 209,620	$ 5,821	0.17	$ 34,241

All costs are in 2021 U.S. dollars and discounted at an annual rate of 3%. Cost-effectiveness ratios may not match previous columns due to rounding.

PDD = patient directed discharges; BUP = buprenorphine; HR = harm reduction; LY = quality-adjusted life year; ICER = incremental cost-effectiveness ratio

Incremental costs, Incremental LYs, and ICER were all compared to the previous relevant scenario (i.e. not dominated).

* PDD discharge rate is set at half of base case.

† Dominated = strategies more costly and less effective than a competing strategy or strategies with an ICER greater than that of a more effective strategy.

‡ These values been rounded to the nearest hundred.

© 2023 Jawa R et al. *JAMA Network Open.*

eTable 3. Cost-effectiveness of Integrated Addiction Services Into Primary Care Assuming Higher Probability of PDD Discharge Rate

Testing Strategy	Cost, $‡	Incremental Cost, $‡	Incremental Life Years (LY) ‡	ICER ($/LY) ‡
**Status Quo**	$ 202,940	--	--	--
**BUP**	$ 209,103	--	--	Dominated**†**
**BUP plus HR**	$ 209,062	$ 6,122	0.17	$ 36,012

All costs are in 2021 U.S. dollars and discounted at an annual rate of 3%. Cost-effectiveness ratios may not match previous columns due to rounding.

PDD = patient directed discharges; BUP = buprenorphine; HR = harm reduction; LY = quality-adjusted life year; ICER = incremental cost-effectiveness ratio; MOUD = medication for opioid use disorder; ACS = addiction consult service

Incremental costs, Incremental LYs, and ICER were all compared to the previous relevant scenario (i.e. not dominated).

* PDD discharge rate is set at twice of base case.

† Dominated = strategies more costly and less effective than a competing strategy or strategies with an ICER greater than that of a more effective strategy.

‡ These values been rounded to the nearest hundred.

© 2023 Jawa R et al. *JAMA Network Open.*

eTable 4. Cost-effectiveness of Integrated Addiction Services Into Primary Care Assuming a Lower Probability of Spontaneous "Unlinkage" From Outpatient Addiction Without MOUD

**Testing Strategy**	Cost, $‡	Incremental Cost, $‡	Incremental Life Years (LY) ‡	ICER ($/LY) ‡
**Status Quo**	$ 203,630	--	--	--
**BUP**	$ 209,750	--	--	Extended Dominance†
**BUP plus HR**	$ 209,559	$ 5,929	0.17	$ 34,876‡

All costs are in 2021 U.S. dollars and discounted at an annual rate of 3%. Cost-effectiveness ratios may not match previous columns due to rounding.

BUP = buprenorphine; HR = harm reduction; LY = quality-adjusted life year; ICER = incremental cost-effectiveness ratio; MOUD = medication for opioid use disorder

Incremental costs, Incremental LYs, and ICER were all compared to the previous relevant scenario (i.e. not dominated).

* Spontaneous "unlinkage" from outpatient addiction without MOUD rate is set at half of base case.

† Extended dominance = or strategies with an ICER greater than that of a more effective strategy.

‡ These values been rounded to the nearest hundred.

© 2023 Jawa R et al. *JAMA Network Open.*

eTable 5. Cost-effectiveness of Integrated Addiction Services Into Primary Care Assuming Higher Probability of Spontaneous "Unlinkage" From Outpatient Addiction Without MOUD

Testing Strategy	Cost, $‡	Incremental Cost, $‡	Incremental Life Years (LY) ‡	ICER ($/LY) ‡
**Status Quo**	$ 203,398	--	--	--
**BUP**	$ 209,311	--	--	Dominated**†**
**BUP plus HR**	$ 209,289	$ 5,891	0.17	$ 34,653

All costs are in 2021 U.S. dollars and discounted at an annual rate of 3%. Cost-effectiveness ratios may not match previous columns due to rounding.

AMA = against medical advice; BUP = buprenorphine; HR = harm reduction; LY = quality-adjusted life year; ICER = incremental cost-effectiveness ratio; MOUD = medication for opioid use disorder; ACS = addiction consult service

Incremental costs, Incremental LYs, and ICER were all compared to the previous relevant scenario (i.e. not dominated).

* Spontaneous "unlinkage" from outpatient addiction without MOUD rate is set at twice of base case.

† Dominated = strategies more costly and less effective than a competing strategy or strategies with an ICER greater than that of a more effective strategy.

‡ These values been rounded to the nearest hundred.

© 2023 Jawa R et al. *JAMA Network Open.*

eTable 6. Cost-effectiveness of Integrated Addiction Services Into Primary Care Assuming a Lower Probability of Hospitalization for Overdose

Testing Strategy	Cost, $‡	Incremental Cost, $‡	Incremental Life Years (LY) ‡	ICER ($/LY) ‡
**Status Quo**	$ 203,505	--	--	--
**BUP**	$ 209,380	--	--	Dominated†
**BUP plus HR**	$ 209,359	$ 5,854	0.17	$ 34,435

All costs are in 2021 U.S. dollars and discounted at an annual rate of 3%. Cost-effectiveness ratios may not match previous columns due to rounding.

BUP = buprenorphine; HR = harm reduction; LY = quality-adjusted life year; ICER = incremental cost-effectiveness ratio

Incremental costs, Incremental LYs, and ICER were all compared to the previous relevant scenario (i.e. not dominated).

* Hospitalization for overdose rate is set at its lowest bound

† Dominated = strategies more costly and less effective than a competing strategy or strategies with an ICER greater than that of a more effective strategy.

‡ These values been rounded to the nearest hundred.

© 2023 Jawa R et al. *JAMA Network Open.*

eTable 7. Cost-effectiveness of Integrated Addiction Services Into Primary Care Assuming a Higher Probability of Hospitalization for Overdose

**Testing Strategy**	Cost, $‡	Incremental Cost, $‡	Incremental Life Years (LY) ‡	ICER ($/LY) ‡
**Status Quo**	$ 203,569	--	--	--
**BUP**	$ 209,437	--	--	Dominated†
**BUP plus HR**	$ 209,416	$ 5,847	0.17	$ 34,394

All costs are in 2021 U.S. dollars and discounted at an annual rate of 3%. Cost-effectiveness ratios may not match previous columns due to rounding.

BUP = buprenorphine; HR = harm reduction; LY = quality-adjusted life year; ICER = incremental cost-effectiveness ratio

Incremental costs, Incremental LYs, and ICER were all compared to the previous relevant scenario (i.e. not dominated).

* Hospitalization for overdose rate is set at its highest bound

† Dominated = strategies more costly and less effective than a competing strategy or strategies with an ICER greater than that of a more effective strategy.

‡ These values been rounded to the nearest hundred.

© 2023 Jawa R et al. *JAMA Network Open.*

eTable 8. Cost-effectiveness of Integrated Addiction Services Into Primary Care Assuming a Lower Probability of Inpatient Linkage

Testing Strategy	Cost, $‡	Incremental Cost, $‡	Incremental Life Years (LY) ‡	ICER ($/LY) ‡
**Status Quo**	$ 202,616	--	--	--
**BUP**	$ 208,952	--	--	Dominated†
**BUP plus HR**	$ 208,793	$ 6,177	0.17	$ 36,335

All costs are in 2021 U.S. dollars and discounted at an annual rate of 3%. Cost-effectiveness ratios may not match previous columns due to rounding.

BUP = buprenorphine; HR = harm reduction; LY = quality-adjusted life year; ICER = incremental cost-effectiveness ratio

Incremental costs, Incremental LYs, and ICER were all compared to the previous relevant scenario (i.e. not dominated).

* Inpatient linkage rate is set at its lowest bound

† Dominated = strategies more costly and less effective than a competing strategy or strategies with an ICER greater than that of a more effective strategy.

‡ These values been rounded to the nearest hundred.

eTable 9. Cost-effectiveness of Integrated Addiction Services Into Primary Care Assuming a Higher Probability of Inpatient Linkage

Testing Strategy	Cost, $‡	Incremental Cost, $‡	Incremental Life Years (LY) ‡	ICER ($/LY) ‡
**Status Quo**	$ 204,287	--	--	--
**BUP**	$ 209,962	--	--	Dominated†
**BUP plus HR**	$ 209,868	$ 5,581	0.16	$ 34,881

All costs are in 2021 U.S. dollars and discounted at an annual rate of 3%. Cost-effectiveness ratios may not match previous columns due to rounding.

BUP = buprenorphine; HR = harm reduction; LY = quality-adjusted life year; ICER = incremental cost-effectiveness ratio

Incremental costs, Incremental LYs, and ICER were all compared to the previous relevant scenario (i.e. not dominated).

* Inpatient linkage rate is set at its highest bound

† Dominated = strategies more costly and less effective than a competing strategy or strategies with an ICER greater than that of a more effective strategy.

‡ These values been rounded to the nearest hundred.

© 2023 Jawa R et al. *JAMA Network Open.*

eTable 10. Cost-effectiveness of Integrated Addiction Services Into Primary Care Assuming a Lower Probability of Fatal Overdose

**Testing Strategy**	Cost, $‡	Incremental Cost, $‡	Incremental Life Years (LY) ‡	ICER ($/LY) ‡
**Status Quo**	$ 204,080	--	--	--
**BUP**	$ 209,969	--	--	Dominated†
**BUP plus HR**	$ 209,875	$ 5,795	0.16	$ 36,219

All costs are in 2021 U.S. dollars and discounted at an annual rate of 3%. Cost-effectiveness ratios may not match previous columns due to rounding.

BUP = buprenorphine; HR = harm reduction; LY = quality-adjusted life year; ICER = incremental cost-effectiveness ratio

Incremental costs, Incremental LYs, and ICER were all compared to the previous relevant scenario (i.e. not dominated).

* Fatal overdose rate is set at its lowest bound

† Dominated = strategies more costly and less effective than a competing strategy or strategies with an ICER greater than that of a more effective strategy.

‡ These values been rounded to the nearest hundred.

© 2023 Jawa R et al. *JAMA Network Open.*

eTable 11. Cost-effectiveness of Integrated Addiction Services Into Primary Care Assuming a Higher Probability of Fatal Overdose

**Testing Strategy**	Cost, $‡	Incremental Cost, $‡	Incremental Life Years (LY) ‡	ICER ($/LY) ‡
**Status Quo**	$ 197,749	--	--	--
**BUP**	$ 204,346	--	--	Dominated**†**
**BUP plus HR**	$ 204,252	$ 6,503	0.19	$ 34,226

All costs are in 2021 U.S. dollars and discounted at an annual rate of 3%. Cost-effectiveness ratios may not match previous columns due to rounding.

BUP = buprenorphine; HR = harm reduction; LY = quality-adjusted life year; ICER = incremental cost-effectiveness ratio

Incremental costs, Incremental LYs, and ICER were all compared to the previous relevant scenario (i.e. not dominated).

* Fatal overdose rate is set at its highest bound

† Dominated = strategies more costly and less effective than a competing strategy or strategies with an ICER greater than that of a more effective strategy.

‡ These values been rounded to the nearest hundred.

© 2023 Jawa R et al. *JAMA Network Open.*

eTable 12. Cost-effectiveness of Integrated Addiction Services Into Primary Care Assuming a Lower Probability Spontaneous "Unlinkage" From Outpatient Addiction With MOUD

**Testing Strategy**	Cost, $‡	Incremental Cost, $‡	Incremental Life Years (LY) ‡	ICER ($/LY) ‡
**Status Quo**	$ 204,355	--	--	--
**BUP**	$ 211,039	--	--	Dominated†
**BUP plus HR**	$ 211,025	$ 6,670	0.18	$ 37,055

All costs are in 2021 U.S. dollars and discounted at an annual rate of 3%. Cost-effectiveness ratios may not match previous columns due to rounding.

BUP = buprenorphine; HR = harm reduction; LY = quality-adjusted life year; ICER = incremental cost-effectiveness ratio; MOUD = medication for opioid use disorder

Incremental costs, Incremental LYs, and ICER were all compared to the previous relevant scenario (i.e. not dominated).

* Spontaneous "unlinkage" from outpatient addiction with MOUD rate is set at half of base case.

† Dominated = strategies more costly and less effective than a competing strategy or strategies with an ICER greater than that of a more effective strategy.

‡ These values been rounded to the nearest hundred.

© 2023 Jawa R et al. *JAMA Network Open.*

eTable 13. Cost-effectiveness of Integrated Addiction Services Into Primary Care Assuming a Higher Probability of Spontaneous "Unlinkage" From Outpatient Addiction With MOUD

Testing Strategy	Cost, $‡	Incremental Cost, $‡	Incremental Life Years (LY) ‡	ICER ($/LY) ‡
**Status Quo**	$ 203,060	--	--	--
**BUP**	$ 208,502	--	--	Dominated†
**BUP plus HR**	$ 208,444	$ 5,384	0.15	$ 35,893

All costs are in 2021 U.S. dollars and discounted at an annual rate of 3%. Cost-effectiveness ratios may not match previous columns due to rounding.

BUP = buprenorphine; HR = harm reduction; LY = quality-adjusted life year; ICER = incremental cost-effectiveness ratio; MOUD = medication for opioid use disorder

Incremental costs, Incremental LYs, and ICER were all compared to the previous relevant scenario (i.e. not dominated).

* Spontaneous "unlinkage" from outpatient addiction with MOUD rate is set at twice of base case.

† Dominated = strategies more costly and less effective than a competing strategy or strategies with an ICER greater than that of a more effective strategy.

‡ These values been rounded to the nearest hundred.

© 2023 Jawa R et al. *JAMA Network Open.*

eTable 14. Cost-effectiveness of Integrated Addiction Services Into Primary Care Assuming a Lower Inpatient MOUD Cost

Testing Strategy	Cost, $‡	Incremental Cost, $‡	Incremental Life Years (LY) ‡	ICER ($/LY) ‡
**Status Quo**	$ 203,537	--	--	--
**BUP**	$ 209,408	--	--	Dominated†
**BUP plus HR**	$ 209,387	$ 5,850	0.17	$ 34,412

All costs are in 2021 U.S. dollars and discounted at an annual rate of 3%. Cost-effectiveness ratios may not match previous columns due to rounding.

BUP = buprenorphine; HR = harm reduction; LY = quality-adjusted life year; ICER = incremental cost-effectiveness ratio; MOUD = medication for opioid use disorder

Incremental costs, Incremental LYs, and ICER were all compared to the previous relevant scenario (i.e. not dominated).

* Inpatient MOUD cost is set at half of base case.

† Dominated = strategies more costly and less effective than a competing strategy or strategies with an ICER greater than that of a more effective strategy.

‡ These values been rounded to the nearest hundred.

© 2023 Jawa R et al. *JAMA Network Open.*

eTable 15. Cost-effectiveness of Integrated Addiction Services Into Primary Care Assuming a Higher Inpatient MOUD Cost

Testing Strategy	Cost, $‡	Incremental Cost, $‡	Incremental Life Years (LY) ‡	ICER ($/LY) ‡
**Status Quo**	$ 203,537	--	--	--
**BUP**	$ 209,408	--	--	Dominated†
**BUP plus HR**	$ 209,387	$ 5,850	0.17	$ 34,412

All costs are in 2021 U.S. dollars and discounted at an annual rate of 3%. Cost-effectiveness ratios may not match previous columns due to rounding.

BUP = buprenorphine; HR = harm reduction; LY = quality-adjusted life year; ICER = incremental cost-effectiveness ratio; MOUD = medication for opioid use disorder

Incremental costs, Incremental LYs, and ICER were all compared to the previous relevant scenario (i.e. not dominated).

* Inpatient MOUD cost is set at twice of base case.

† Dominated = strategies more costly and less effective than a competing strategy or strategies with an ICER greater than that of a more effective strategy.

‡ These values been rounded to the nearest hundred.

© 2023 Jawa R et al. *JAMA Network Open.*

eTable 16. Cost-effectiveness of Integrated Addiction Services Into Primary Care Assuming a Lower Previous Infection Multiplier for Risk of Subsequent Infection

Testing Strategy	Cost, $‡	Incremental Cost, $‡	Incremental Life Years (LY) ‡	ICER ($/LY) ‡
**Status Quo**	$ 203,264	--	--	--
**BUP**	$ 208,978	--	--	Dominated†
**BUP plus HR**	$ 208,903	$ 5,639	0.16	$ 35,244

All costs are in 2021 U.S. dollars and discounted at an annual rate of 3%. Cost-effectiveness ratios may not match previous columns due to rounding.

BUP = buprenorphine; HR = harm reduction; LY = quality-adjusted life year; ICER = incremental cost-effectiveness ratio

Incremental costs, Incremental LYs, and ICER were all compared to the previous relevant scenario (i.e. not dominated).

* Previous infection multiplier for risk of subsequent infection is set at its lowest bound.

† Dominated = strategies more costly and less effective than a competing strategy or strategies with an ICER greater than that of a more effective strategy.

‡ These values been rounded to the nearest hundred.

© 2023 Jawa R et al. *JAMA Network Open.*

eTable 17. Cost-effectiveness of Integrated Addiction Services Into Primary Care Assuming a Higher Previous Infection Multiplier for Risk of Subsequent Infection

Testing Strategy	Cost, $‡	Incremental Cost, $‡	Incremental Life Years (LY) ‡	ICER ($/LY) ‡
**Status Quo**	$ 204,485	--	--	--
**BUP**	$ 211,644	--	--	Dominated†
**BUP plus HR**	$ 209,387	$ 4,902	0.29	$ 16,903

All costs are in 2021 U.S. dollars and discounted at an annual rate of 3%. Cost-effectiveness ratios may not match previous columns due to rounding.

BUP = buprenorphine; HR = harm reduction; LY = quality-adjusted life year; ICER = incremental cost-effectiveness ratio

Incremental costs, Incremental LYs, and ICER were all compared to the previous relevant scenario (i.e. not dominated).

* Previous infection multiplier for risk of subsequent infection is set at its highest bound.

† Dominated = strategies more costly and less effective than a competing strategy or strategies with an ICER greater than that of a more effective strategy.

‡ These values been rounded to the nearest hundred.

© 2023 Jawa R et al. *JAMA Network Open.*

eTable 18. Cost-effectiveness of Integrated Addiction Services Into Primary Care Assuming a Lower Probability of Background or Inpatient Linkage of Outpatient Addiction Care

**Testing Strategy**	Cost, $‡	Incremental Cost, $‡	Incremental Life Years (LY) ‡	ICER ($/LY) ‡
**Status Quo**	$ 202,893	--	--	--
**BUP plus HR**	$ 206,434	$3,450	0.097	$ 35,570
**BUP**	$ 208,725	$2,382	0.036	$ 66,040

All costs are in 2021 U.S. dollars and discounted at an annual rate of 3%. Cost-effectiveness ratios may not match previous columns due to rounding.

BUP = buprenorphine; HR = harm reduction; LY = quality-adjusted life year; ICER = incremental cost-effectiveness ratio; ACS = addiction consult service

Incremental costs, Incremental LYs, and ICER were all compared to the previous relevant scenario (i.e. not dominated).

* Background or inpatient linkage of o**utpatient addiction care** rate is set at half of basecase.

‡ These values been rounded to the nearest hundred.

© 2023 Jawa R et al. *JAMA Network Open.*

eTable 19. Cost-effectiveness of Integrated Addiction Services Into Primary Care Assuming a Higher Probability of Background or Inpatient Linkage of Outpatient Addiction Care

**Testing Strategy**	Cost, $‡	Incremental Cost, $‡	Incremental Life Years (LY) ‡	ICER ($/LY) ‡
**Status Quo**	$ 204,564	--	--	--
**BUP**	$ 211,013	--	--	Extended dominance†
**BUP plus HR**	$ 211,063	$ 6,499	0.2	$ 32,495

All costs are in 2021 U.S. dollars and discounted at an annual rate of 3%. Cost-effectiveness ratios may not match previous columns due to rounding.

BUP = buprenorphine; HR = harm reduction; LY = quality-adjusted life year; ICER = incremental cost-effectiveness ratio; ACS = addiction consult service

Incremental costs, Incremental LYs, and ICER were all compared to the previous relevant scenario (i.e. not dominated).

* Background or inpatient linkage of o**utpatient addiction care** rate is set at twice of basecase.

† Extended dominance = or strategies with an ICER greater than that of a more effective strategy.

‡ These values been rounded to the nearest hundred.

© 2023 Jawa R et al. *JAMA Network Open.*

eTable 20. Cost-effectiveness of Integrated Addiction Services Into Primary Care Assuming Lower Probability of Needle Sharing

**Testing Strategy**	Cost, $‡	Incremental Cost, $‡	Incremental Life Years (LY) ‡	ICER ($/LY) ‡
**Status Quo**	$ 203,411	--	--	--
**BUP**	$ 209,234	--	--	Dominated†
**BUP plus HR**	$ 209,158	$ 5,747	0.16	$ 35,919

All costs are in 2021 U.S. dollars and discounted at an annual rate of 3%. Cost-effectiveness ratios may not match previous columns due to rounding.

BUP = buprenorphine; HR = harm reduction; LY = quality-adjusted life year; ICER = incremental cost-effectiveness ratio

Incremental costs, Incremental LYs, and ICER were all compared to the previous relevant scenario (i.e. not dominated).

* Needle sharing rate is set at half of base case.

† Dominated = strategies more costly and less effective than a competing strategy or strategies with an ICER greater than that of a more effective strategy.

‡ These values been rounded to the nearest hundred.

eTable 21. Cost-effectiveness of Integrated Addiction Services Into Primary Care Assuming a Higher Probability of Needle Sharing

Testing Strategy	Cost, $‡	Incremental Cost, $‡	Incremental Life Years (LY) ‡	ICER ($/LY) ‡
**Status Quo**	$ 203,714	--	--	--
**BUP**	$ 209,877	--	--	Dominated†
**BUP plus HR**	$ 209,789	$ 6,075	0.17	$ 35,735

All costs are in 2021 U.S. dollars and discounted at an annual rate of 3%. Cost-effectiveness ratios may not match previous columns due to rounding.

BUP = buprenorphine; HR = harm reduction; LY = quality-adjusted life year; ICER = incremental cost-effectiveness ratio

Incremental costs, Incremental LYs, and ICER were all compared to the previous relevant scenario (i.e. not dominated).

* Needle sharing rate is set at twice of base case.

† Dominated = strategies more costly and less effective than a competing strategy or strategies with an ICER greater than that of a more effective strategy.

‡ These values been rounded to the nearest hundred.

© 2023 Jawa R et al. *JAMA Network Open.*

eTable 22. Cost-effectiveness of Integrated Addiction Services Into Primary Care Assuming a Lower Probability of Death, Inpatient With SSTI

**Testing Strategy**	Cost, $‡	Incremental Cost, $‡	Incremental Life Years (LY) ‡	ICER ($/LY) ‡
**Status Quo**	$ 203,498	--	--	--
**BUP**	$ 209,477	--	--	Dominated†
**BUP plus HR**	$ 209,459	$ 5,961	0.17	$ 35,064

All costs are in 2021 U.S. dollars and discounted at an annual rate of 3%. Cost-effectiveness ratios may not match previous columns due to rounding.

BUP = buprenorphine; HR = harm reduction; LY = quality-adjusted life year; ICER = incremental cost-effectiveness ratio; SSTI = skin and soft tissue infection

Incremental costs, Incremental LYs, and ICER were all compared to the previous relevant scenario (i.e. not dominated).

* probability of death, inpatient with SSTI rate is set at its lowest bound

† Dominated = strategies more costly and less effective than a competing strategy or strategies with an ICER greater than that of a more effective strategy.

‡ These values been rounded to the nearest hundred.

© 2023 Jawa R et al. *JAMA Network Open.*

eTable 23. Cost-effectiveness of Integrated Addiction Services Into Primary Care Assuming a Higher Probability of Death, Inpatient With SSTI

**Testing Strategy**	Cost, $‡	Incremental Cost, $‡	Incremental Life Years (LY) ‡	ICER ($/LY) ‡
**Status Quo**	$ 203,408	--	--	--
**BUP**	$ 209,318	--	--	Dominated†
**BUP plus HR**	$ 209,260	$ 5,851	0.17	$ 34,418

All costs are in 2021 U.S. dollars and discounted at an annual rate of 3%. Cost-effectiveness ratios may not match previous columns due to rounding.

BUP = buprenorphine; HR = harm reduction; LY = quality-adjusted life year; ICER = incremental cost-effectiveness ratio; SSTI = skin and soft tissue infection

Incremental costs, Incremental LYs, and ICER were all compared to the previous relevant scenario (i.e. not dominated).

* probability of death, inpatient with SSTI rate is set at its highest bound

† Dominated = strategies more costly and less effective than a competing strategy or strategies with an ICER greater than that of a more effective strategy.

‡ These values been rounded to the nearest hundred.

© 2023 Jawa R et al. *JAMA Network Open.*

eTable 24. Cost-effectiveness of Integrated Addiction Services Into Primary Care Assuming a Lower Probability of Hospitalization For SSTI

**Testing Strategy**	Cost, $‡	Incremental Cost, $‡	Incremental Life Years (LY) ‡	ICER ($/LY) ‡
**Status Quo**	$ 200,619	--	--	--
**BUP**	$ 205,274	--	--	Extended Dominance†
**BUP plus HR**	$ 205,344	$ 4,724	0.167	$ 28,211

All costs are in 2021 U.S. dollars and discounted at an annual rate of 3%. Cost-effectiveness ratios may not match previous columns due to rounding.

BUP = buprenorphine; HR = harm reduction; LY = quality-adjusted life year; ICER = incremental cost-effectiveness ratio; SSTI = skin and soft tissue infection

Incremental costs, Incremental LYs, and ICER were all compared to the previous relevant scenario (i.e. not dominated).

* probability of hospitalization for SSTI rate is set at its lowest bound

† Extended dominance = or strategies with an ICER greater than that of a more effective strategy.

‡ These values been rounded to the nearest hundred.

© 2023 Jawa R et al. *JAMA Network Open.*

eTable 25. Cost-effectiveness of Integrated Addiction Services Into Primary Care Assuming a Higher Probability of Hospitalization for SSTI

**Testing Strategy**	Cost, $‡	Incremental Cost, $‡	Incremental Life Years (LY) ‡	ICER ($/LY) ‡
**Status Quo**	$ 206,020	--	--	--
**BUP**	$ 212,691	--	--	Dominated†
**BUP plus HR**	$ 212,596	$ 6,576	0.17	$ 38,682

All costs are in 2021 U.S. dollars and discounted at an annual rate of 3%. Cost-effectiveness ratios may not match previous columns due to rounding.

BUP = buprenorphine; HR = harm reduction; LY = quality-adjusted life year; ICER = incremental cost-effectiveness ratio; SSTI = skin and soft tissue infection

Incremental costs, Incremental LYs, and ICER were all compared to the previous relevant scenario (i.e. not dominated).

* probability of hospitalization for SSTI rate is set at its highest bound

† Dominated = strategies more costly and less effective than a competing strategy or strategies with an ICER greater than that of a more effective strategy.

‡ These values been rounded to the nearest hundred.

© 2023 Jawa R et al. *JAMA Network Open.*

eTable 26. Cost-effectiveness of Integrated Addiction Services Into Primary Care Assuming a Lower Probability of Hospitalization for IE

Testing Strategy	Cost, $‡	Incremental Cost, $‡	Incremental Life Years (LY) ‡	ICER ($/LY) ‡
**Status Quo**	$ 199,145	--	--	--
**BUP**	$ 206,064	$ 6,919	0.16	$ 43,244
**BUP plus HR**	$ 212,293	$ 6,229	0.006	$ 1,038,166

All costs are in 2021 U.S. dollars and discounted at an annual rate of 3%. Cost-effectiveness ratios may not match previous columns due to rounding.

BUP = buprenorphine; HR = harm reduction; LY = quality-adjusted life year; ICER = incremental cost-effectiveness ratio; IE = infective endocarditis

Incremental costs, Incremental LYs, and ICER were all compared to the previous relevant scenario (i.e. not dominated).

* Hospitalization for IE rate is set at its lowest bound

‡ These values been rounded to the nearest hundred.

© 2023 Jawa R et al. *JAMA Network Open.*

eTable 27. Cost-effectiveness of Integrated Addiction Services Into Primary Care Assuming a Higher Probability of Hospitalization for IE

Testing Strategy	Cost, $‡	Incremental Cost, $‡	Incremental Life Years (LY) ‡	ICER ($/LY) ‡
**Status Quo**	$ 207,621	--	--	--
**BUP**	$ 212,378	--	--	Dominated**†**
**BUP plus HR**	$ 212,293	$ 4,672	0.17	$ 27,482

All costs are in 2021 U.S. dollars and discounted at an annual rate of 3%. Cost-effectiveness ratios may not match previous columns due to rounding.

BUP = buprenorphine; HR = harm reduction; LY = quality-adjusted life year; ICER = incremental cost-effectiveness ratio; IE = infective endocarditis

Incremental costs, Incremental LYs, and ICER were all compared to the previous relevant scenario (i.e. not dominated).

* Hospitalization for IE rate is set at its highest bound

† Dominated = strategies more costly and less effective than a competing strategy or strategies with an ICER greater than that of a more effective strategy.

‡ These values been rounded to the nearest hundred.

© 2023 Jawa R et al. *JAMA Network Open.*

eTable 28. Cost-effectiveness of Integrated Addiction Services Into Primary Care Assuming Lower Cost of Untreated Non-Fatal Overdose

Testing Strategy	Cost, $‡	Incremental Cost, $‡	Incremental Life Years (LY) ‡	ICER ($/LY) ‡
**Status Quo**	$ 203,406	--	--	--
**BUP**	$ 209,299	--	--	Dominated†
**BUP plus HR**	$ 209,277	$ 5,871	0.17	$ 34,535

All costs are in 2021 U.S. dollars and discounted at an annual rate of 3%. Cost-effectiveness ratios may not match previous columns due to rounding.

BUP = buprenorphine; HR = harm reduction; LY = quality-adjusted life year; ICER = incremental cost-effectiveness ratio

Incremental costs, Incremental LYs, and ICER were all compared to the previous relevant scenario (i.e. not dominated).

* cost of untreated non-fatal overdose is set at its lowest bound.

† Dominated = strategies more costly and less effective than a competing strategy or strategies with an ICER greater than that of a more effective strategy.

‡ These values been rounded to the nearest hundred.

© 2023 Jawa R et al. *JAMA Network Open.*

eTable 29. Cost-effectiveness of Integrated Addiction Services Into Primary Care Assuming a Higher Cost of Untreated Non-Fatal Overdose

Testing Strategy	Cost, $‡	Incremental Cost, $‡	Incremental Life Years (LY) ‡	ICER ($/LY) ‡
**Status Quo**	$ 203,668	--	--	--
**BUP**	$ 209,519	--	--	Dominated†
**BUP plus HR**	$ 209,497	$ 5,829	0.17	$ 34,288

All costs are in 2021 U.S. dollars and discounted at an annual rate of 3%. Cost-effectiveness ratios may not match previous columns due to rounding.

BUP = buprenorphine; HR = harm reduction; LY = quality-adjusted life year; ICER = incremental cost-effectiveness ratio

Incremental costs, Incremental LYs, and ICER were all compared to the previous relevant scenario (i.e. not dominated).

* cost of untreated non-fatal overdose is set at its highest bound.

† Dominated = strategies more costly and less effective than a competing strategy or strategies with an ICER greater than that of a more effective strategy.

‡ These values been rounded to the nearest hundred.

© 2023 Jawa R et al. *JAMA Network Open.*

eTable 30. Cost-effectiveness of Integrated Addiction Services Into Primary Care Assuming a Lower Fatal Overdose Cost

**Testing Strategy**	Cost, $‡	Incremental Cost, $‡	Incremental Life Years (LY) ‡	ICER ($/LY) ‡
**Status Quo**	$ 203,406	--	--	--
**BUP**	$ 209,299	--	--	Dominated**†**
**BUP plus HR**	$ 209,277	$ 5,871	0.17	$ 34,535

All costs are in 2021 U.S. dollars and discounted at an annual rate of 3%. Cost-effectiveness ratios may not match previous columns due to rounding.

BUP = buprenorphine; HR = harm reduction; LY = quality-adjusted life year; ICER = incremental cost-effectiveness ratio

Incremental costs, Incremental LYs, and ICER were all compared to the previous relevant scenario (i.e. not dominated).

* cost of fatal overdose is set at its lowest bound.

† Dominated = strategies more costly and less effective than a competing strategy or strategies with an ICER greater than that of a more effective strategy.

‡ These values been rounded to the nearest hundred.

eTable 31. Cost-effectiveness of Integrated Addiction Services Into Primary Care Assuming Higher Fatal Overdose Cost

Testing Strategy	Cost, $‡	Incremental Cost, $‡	Incremental Life Years (LY) ‡	ICER ($/LY) ‡
**Status Quo**	$ 203,668	--	--	--
**BUP**	$ 209,519	--	--	Dominated**†**
**BUP plus HR**	$ 209,497	$ 5,859	0.17	$ 34,288

All costs are in 2021 U.S. dollars and discounted at an annual rate of 3%. Cost-effectiveness ratios may not match previous columns due to rounding.

BUP = buprenorphine; HR = harm reduction; LY = quality-adjusted life year; ICER = incremental cost-effectiveness ratio

Incremental costs, Incremental LYs, and ICER were all compared to the previous relevant scenario (i.e. not dominated).

* cost of fatal overdose is set at its highest bound.

† Dominated = strategies more costly and less effective than a competing strategy or strategies with an ICER greater than that of a more effective strategy.

‡ These values been rounded to the nearest hundred.

© 2023 Jawa R et al. *JAMA Network Open.*

eTable 32. Cost-effectiveness of Integrated Addiction Services Into Primary Care Assuming a Lower Probability of Death From Untreated SSTI

**Testing Strategy**	Cost, $‡	Incremental Cost, $‡	Incremental Life Years (LY) ‡	ICER ($/LY) ‡
**Status Quo**	$ 203,537	--	--	--
**BUP**	$ 209,408	--	--	Dominated†
**BUP plus HR**	$ 209,387	$ 5,850	0.17	$ 34,412

All costs are in 2021 U.S. dollars and discounted at an annual rate of 3%. Cost-effectiveness ratios may not match previous columns due to rounding.

BUP = buprenorphine; HR = harm reduction; LY = quality-adjusted life year; ICER = incremental cost-effectiveness ratio; SSTI = skin and soft tissue infection

Incremental costs, Incremental LYs, and ICER were all compared to the previous relevant scenario (i.e. not dominated).

* probability of death from untreated SSTI rate is set at its lowest bound.

† Dominated = strategies more costly and less effective than a competing strategy or strategies with an ICER greater than that of a more effective strategy.

‡ These values been rounded to the nearest hundred.

© 2023 Jawa R et al. *JAMA Network Open.*

eTable 33. Cost-effectiveness of Integrated Addiction Services Into Primary Care Assuming a Higher Probability of Death From Untreated SSTI

Testing Strategy	Cost, $‡	Incremental Cost, $‡	Incremental Life Years (LY) ‡	ICER ($/LY) ‡
**Status Quo**	$ 194,393	--	--	--
**BUP**	$ 205,087	--	--	Extended Dominance†
**BUP plus HR**	$ 205,106	$ 10,713	0.3	$ 35,480

All costs are in 2021 U.S. dollars and discounted at an annual rate of 3%. Cost-effectiveness ratios may not match previous columns due to rounding.

BUP = buprenorphine; HR = harm reduction; LY = quality-adjusted life year; ICER = incremental cost-effectiveness ratio; SSTI = skin and soft tissue infection

Incremental costs, Incremental LYs, and ICER were all compared to the previous relevant scenario (i.e. not dominated).

* probability of death from untreated SSTI rate is set at its highest bound.

† Extended dominance = or strategies with an ICER greater than that of a more effective strategy.

‡ These values been rounded to the nearest hundred.

© 2023 Jawa R et al. *JAMA Network Open.*

eTable 34. Cost-effectiveness of Integrated Addiction Services Into Primary Care Assuming a Lower Probability of Death From Untreated IE

Testing Strategy	Cost, $‡	Incremental Cost, $‡	Incremental Life Years (LY) ‡	ICER ($/LY) ‡
**Status Quo**	$ 203,537	--	--	--
**BUP**	$ 209,408	--	--	Dominated†
**BUP plus HR**	$ 209,387	$ 5,850	0.17	$ 34,412

All costs are in 2021 U.S. dollars and discounted at an annual rate of 3%. Cost-effectiveness ratios may not match previous columns due to rounding.

BUP = buprenorphine; HR = harm reduction; LY = quality-adjusted life year; ICER = incremental cost-effectiveness ratio; IE = infective endocarditis

Incremental costs, Incremental LYs, and ICER were all compared to the previous relevant scenario (i.e. not dominated).

* probability of death, untreated IE rate is set at its lowest bound.

† Dominated = strategies more costly and less effective than a competing strategy or strategies with an ICER greater than that of a more effective strategy.

‡ These values been rounded to the nearest hundred.

© 2023 Jawa R et al. *JAMA Network Open.*

eTable 35. Cost-effectiveness of Integrated Addiction Services Into Primary Care Assuming a Higher Probability of Death From Untreated IE

Testing Strategy	Cost, $‡	Incremental Cost, $‡	Incremental Life Years (LY) ‡	ICER ($/LY) ‡
**Status Quo**	$ 192,654	--	--	--
**BUP**	$ 201,244	--	--	Extended Dominance†
**BUP plus HR**	$ 201,468	$ 8,814	0.21	$ 40,955

All costs are in 2021 U.S. dollars and discounted at an annual rate of 3%. Cost-effectiveness ratios may not match previous columns due to rounding.

BUP = buprenorphine; HR = harm reduction; LY = quality-adjusted life year; ICER = incremental cost-effectiveness ratio; IE = infective endocarditis

Incremental costs, Incremental LYs, and ICER were all compared to the previous relevant scenario (i.e. not dominated).

* probability of death, untreated IE rate is set at its highest bound.

† Extended dominance = or strategies with an ICER greater than that of a more effective strategy.

‡ These values been rounded to the nearest hundred.

eTable 36. Cost-effectiveness of Integrated Addiction Services Into Primary Care Assuming a Lower Cost of Outpatient Addiction Visit With MOUD

Testing Strategy	Cost, $‡	Incremental Cost, $‡	Incremental Life Years (LY) ‡	ICER ($/LY) ‡
**Status Quo**	$ 203,021	--	--	--
**BUP**	$ 208,260	--	--	Dominated†
**BUP plus HR**	$ 208,167	$ 5,146	0.17	$ 30,271

All costs are in 2021 U.S. dollars and discounted at an annual rate of 3%. Cost-effectiveness ratios may not match previous columns due to rounding.

BUP = buprenorphine; HR = harm reduction; LY = quality-adjusted life year; ICER = incremental cost-effectiveness ratio; MOUD = medication for opioid use disorder

Incremental costs, Incremental LYs, and ICER were all compared to the previous relevant scenario (i.e. not dominated).

* cost of outpatient addiction visit with MOUD is set at half of base case.

† Dominated = strategies more costly and less effective than a competing strategy or strategies with an ICER greater than that of a more effective strategy.

‡ These values been rounded to the nearest hundred.

© 2023 Jawa R et al. *JAMA Network Open.*

eTable 37. Cost-effectiveness of Integrated Addiction Services Into Primary Care Assuming Higher Cost of Outpatient Addiction Visit With MOUD

Testing Strategy	Cost, $‡	Incremental Cost, $‡	Incremental Life Years (LY) ‡	ICER ($/LY) ‡
**Status Quo**	$ 204,568	--	--	--
**BUP**	$ 211,704	--	--	Extended Dominance†
**BUP plus HR**	$ 211,828	$ 7,259	0.167	$ 43,348

All costs are in 2021 U.S. dollars and discounted at an annual rate of 3%. Cost-effectiveness ratios may not match previous columns due to rounding.

BUP = buprenorphine; HR = harm reduction; LY = quality-adjusted life year; ICER = incremental cost-effectiveness ratio; MOUD = medication for opioid use disorder

Incremental costs, Incremental LYs, and ICER were all compared to the previous relevant scenario (i.e. not dominated).

* cost of outpatient addiction visit with MOUD is set at twice of base case.

† Extended dominance = or strategies with an ICER greater than that of a more effective strategy.

‡ These values been rounded to the nearest hundred.

© 2023 Jawa R et al. *JAMA Network Open.*

eTable 38. Cost-effectiveness of Integrated Addiction Services Into Primary Care Assuming a Lower Probability of Outpatient Addiction Care From Inpatient Care (No MOUD) Without ACS

Testing Strategy	Cost, $‡	Incremental Cost, $‡	Incremental Life Years (LY) ‡	ICER ($/LY) ‡
**Status Quo**	$ 200,889	--	--	--
**BUP**	$ 206,327	--	--	Extended Dominance†
**BUP plus HR**	$ 206,343	$ 5,454	0.13	$ 41,954

All costs are in 2021 U.S. dollars and discounted at an annual rate of 3%. Cost-effectiveness ratios may not match previous columns due to rounding.

BUP = buprenorphine; HR = harm reduction; LY = quality-adjusted life year; ICER = incremental cost-effectiveness ratio; ACS = addiction consult service; MOUD = medication for opioid use disorder

* outpatient addiction care from inpatient care (No MOUD) without ACS rate is set at half of base case.

† Extended dominance = or strategies with an ICER greater than that of a more effective strategy.

‡These values are not the same, but have been rounded to the nearest hundred

© 2023 Jawa R et al. *JAMA Network Open.*

eTable 39. Cost-effectiveness of Integrated Addiction Services Into Primary Care Assuming a Higher Probability of Outpatient Addiction Care From Inpatient Care (No MOUD) Without ACS

Testing Strategy	Cost, $‡	Incremental Cost, $‡	Incremental Life Years (LY) ‡	ICER ($/LY) ‡
**Status Quo**	$ 209,121	--	--	--
**BUP**	$ 212,362	--	--	Dominated†
**BUP plus HR**	$ 212,233	$ 3,112	0.14	$ 22,229

All costs are in 2021 U.S. dollars and discounted at an annual rate of 3%. Cost-effectiveness ratios may not match previous columns due to rounding.

BUP = buprenorphine; HR = harm reduction; LY = quality-adjusted life year; ICER = incremental cost-effectiveness ratio; ACS = addiction consult service; MOUD = medication for opioid use disorder

* outpatient addiction care from inpatient care (No MOUD) without ACS rate is set at twice of base case.

† Dominated = strategies more costly and less effective than a competing strategy or strategies with an ICER greater than that of a more effective strategy.

‡These values are not the same, but have been rounded to the nearest hundred

© 2023 Jawa R et al. *JAMA Network Open.*

eTable 40. Cost-effectiveness of Integrated Addiction Services Into Primary Care Assuming a Lower Probability of Linkage to Outpatient Addiction Care (MOUD) From Inpatient Without ACS, With MOUD

Testing Strategy	Cost, $‡	Incremental Cost, $‡	Incremental Life Years (LY) ‡	ICER ($/LY) ‡
**Status Quo**	$ 203,537	--	--	--
**BUP**	$ 209,409	--	--	Dominated†
**BUP plus HR**	$ 209,387	$ 5,850	0.17	$ 34,412

All costs are in 2021 U.S. dollars and discounted at an annual rate of 3%. Cost-effectiveness ratios may not match previous columns due to rounding.

BUP = buprenorphine; HR = harm reduction; LY = quality-adjusted life year; ICER = incremental cost-effectiveness ratio; ACS = addiction consult service; MOUD = medication for opioid use disorder

* Outpatient addiction care (MOUD) from inpatient without ACS, with MOUD rate is set at its lowest bound

† Dominated = strategies more costly and less effective than a competing strategy or strategies with an ICER greater than that of a more effective strategy.

‡These values are not the same, but have been rounded to the nearest hundred

© 2023 Jawa R et al. *JAMA Network Open.*

eTable 41. Cost-effectiveness of Integrated Addiction Services Into Primary Care Assuming a Higher Probability of Linkage to Outpatient Addiction Care (MOUD) From Inpatient Without ACS, With MOUD

Testing Strategy	Cost, $‡	Incremental Cost, $‡	Incremental Life Years (LY) ‡	ICER ($/LY) ‡
**Status Quo**	$ 203,537	--	--	--
**BUP**	$ 209,409	--	--	Dominated†
**BUP plus HR**	$ 209,387	$ 5,850	0.17	$ 34,412

All costs are in 2021 U.S. dollars and discounted at an annual rate of 3%. Cost-effectiveness ratios may not match previous columns due to rounding.

BUP = buprenorphine; HR = harm reduction; LY = quality-adjusted life year; ICER = incremental cost-effectiveness ratio; ACS = addiction consult service; MOUD = medication for opioid use disorder

* Outpatient addiction care (MOUD) from inpatient without ACS, with MOUD rate is set at its highest bound

† Dominated = strategies more costly and less effective than a competing strategy or strategies with an ICER greater than that of a more effective strategy.

‡These values are not the same, but have been rounded to the nearest hundred

© 2023 Jawa R et al. *JAMA Network Open.*

eTable 42. Model Parameters, Data Type Used to Inform Parameters, and Sampling Distributions for Probabilistic Sensitivity Analyses

Parameter	Range	PSA Distributions	Source
Population			
Needle sharing prevalence	0.175-0.7	Uniform	[61-65]
Sequelae of Drug use			
Probability of fatal OD	0.1000-0.2200	Uniform	[32-34]
Probability of hospitalization for IE	0.133-0.167	Uniform	[72]
Previous infection multiplier for risk of subsequent infection	1.50-5.10	Uniform	[74]
Inpatient			
Probability of PDD	0.0125-0.0600	Uniform	[75,76]
Outpatient addiction care linkages			
Linkage to outpatient addiction care post hospitalization if on MOUD	0.5-0.99	Uniform	Expert opinion
Linkage to outpatient addiction care, status quo	0.0014646-0.01236164	Uniform	[40,77]
Linkage to outpatient addiction care, BUP	0.0085-0.034	Uniform	[37,77]
Linkage to outpatient addiction care, BUP plus HR	0.0085-0.034	Uniform	[37,77]
Outpatient MOUD initiation			
Outpatient addiction care	0.15-0.6	Uniform	[23]
Outpatient unlinking			
Spontaneous "unlinkage" from outpatient addiction care with MOUD	0.018552-0.074208	Uniform	[78]
Spontaneous "unlinkage" from outpatient addiction without MOUD	0.0619761-0.247942	Uniform	[78]
Mortality			
Probability of death, untreated IE	0.0848-0.5358	Uniform	[79,80]
Probability of death, untreated SSTI	0.00115-0.0028	Uniform	[80]
Probability of death, inpatient with SSTI	0.00035-0.0012	Uniform	[80]
Costs			
Fatal OD	$230.295-$690	Uniform	[87]
NFOD not hospitalized	$598.8-$1798	Uniform	[87]
Hospitalization for IE	$8,736-$34,410	Uniform	[37]
Hospitalization for SSTI	$9,124-$26,378	Uniform	[37]
Hospitalization for OD	$12,744-$15,646	Uniform	[87]
Inpatient MOUD costs	$21.815-$87.26	Uniform	[88]
Outpatient addiction visit with MOUD, status quo	$63.445-$253.78	Uniform	([89], Expert Opinion[b])

© 2023 Jawa R et al. *JAMA Network Open.*

Outpatient addiction visit with MOUD, BUP	$32.32-$129.28	Uniform	([89], Expert Opinion[b])
Outpatient addiction visit with MOUD, BUP plus HR	$34.29-$137.16	Uniform	([89], Expert Opinion[b])

Notes: The ReDUCE Model runs on a weekly time cycle, therefore, all probabilities in this table are weekly probabilities

Calibrated inputs have been adjusted to meet the five calibration points

Abbreviations: PDD=patient directed discharge; BUP= onsite buprenorphine prescribing; BUP plus HR=onsite buprenorphine plus harm reduction; IE=infective endocarditis; MOUD=medication for opioid use disorder; NFOD= nonfatal overdose; OD= overdose; PDD=patient directed discharge; SSTI=severe skin and soft tissue infections

[a,b] Consensus obtained between J.A.B, R.J. and study authors

PSA: We performed probabilistic sensitivity analysis to characterize parameter uncertainty. To address parameter uncertainty and create credible intervals for outcome estimates. The model is programmed to utilize normal, uniform, and lognormal distributions for PSA.

© 2023 Jawa R et al. *JAMA Network Open.*

eTable 43. Model Outcomes and Credible Intervals for Status Quo, BUP, and BUP + HR

Outcomes	Status Quo	Credible Interval*	BUP	Credible Interval*	BUP + HR	Credible Interval*
total cases of fatal OD per 10,000	1,162	1,144-2,303	1,004	802-1,718	1,005	692-1,810
total cases of SSTIs per 10,000	10,846	11,764-25,020	10,943	9,229-15,546	10,682	8,982-15,470
total cases of IE per 10,000	4,185	1,828-5,194	2,897	2,057-5,727	2824	2,068-5,717
total cases of hospitalizations per 10,000	10,957	14,270-28,839	12,412	10,510-16,883	12,127	9,973-16,732
Number of outpatient MOUD initiations per 10,000	14,228	13,728-15,938	63,593	55,444-63,370	63,719	57,180-65,270
IDU Mortality attributable to OD per 10,000	1,180	1,175-2,337	1,019	832-1,731	1,020	720-1,855
IDU Mortality attributable to SSTI per 10,000	625	15-128	230	175-325	225	157-309
IDU Mortality attributable to IE per 10,000	1,629	737-2,024	1,053	804-2,194	1,029	885-2,323
Total hospitalization costs per person	67,192	64,344-142,003	65,440	50,832-101,161	63,860	49,293-100,293
Total outpatient costs per person	43,372	45,537-54,579	104,649	89,055-103,998	111,373	97,552-113,826
Total healthcare costs per person	100,564	109,881-196,582	170,089	139,887-205,159	175,233	146,845-214,119

*95% credible interval was derived from the probabilistic sensitivity analysis. Credible intervals were not calculated for certain outcomes because they are not primary outcomes from the model, but rather were calculated by combining multiple outcomes.

Abbreviations: BUP= onsite buprenorphine prescribing; BUP plus HR=onsite buprenorphine plus harm reduction; IDU= injection drug use; IE=infective endocarditis; MOUD=medication for opioid use disorder; OD= overdose; SSTI=severe skin and soft tissue infections

© 2023 Jawa R et al. *JAMA Network Open.*

eFigure 1. Lifetime Clinical Outcomes Cases Averted per 10,000 in BUP, BUP + HR Compared to Status Quo Strategies

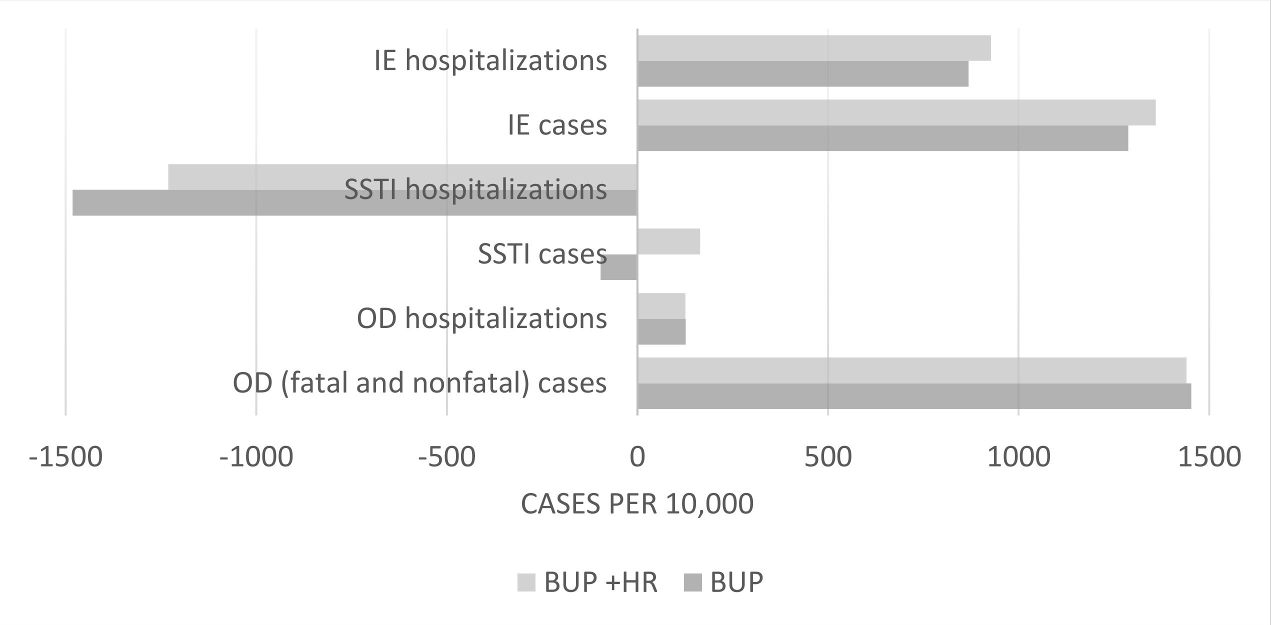

IE=infective endocarditis; SSTI=severe skin and soft tissue infections; OD= overdose; BUP = Onsite buprenorphine; BUP+HR = Onsite buprenorphine and harm reduction; SQ= status quo

© 2023 Jawa R et al. *JAMA Network Open.*

eFigure 2. Deterministic Sensitivity Analysis- Tornado Chart of BUP + HR Versus SQ

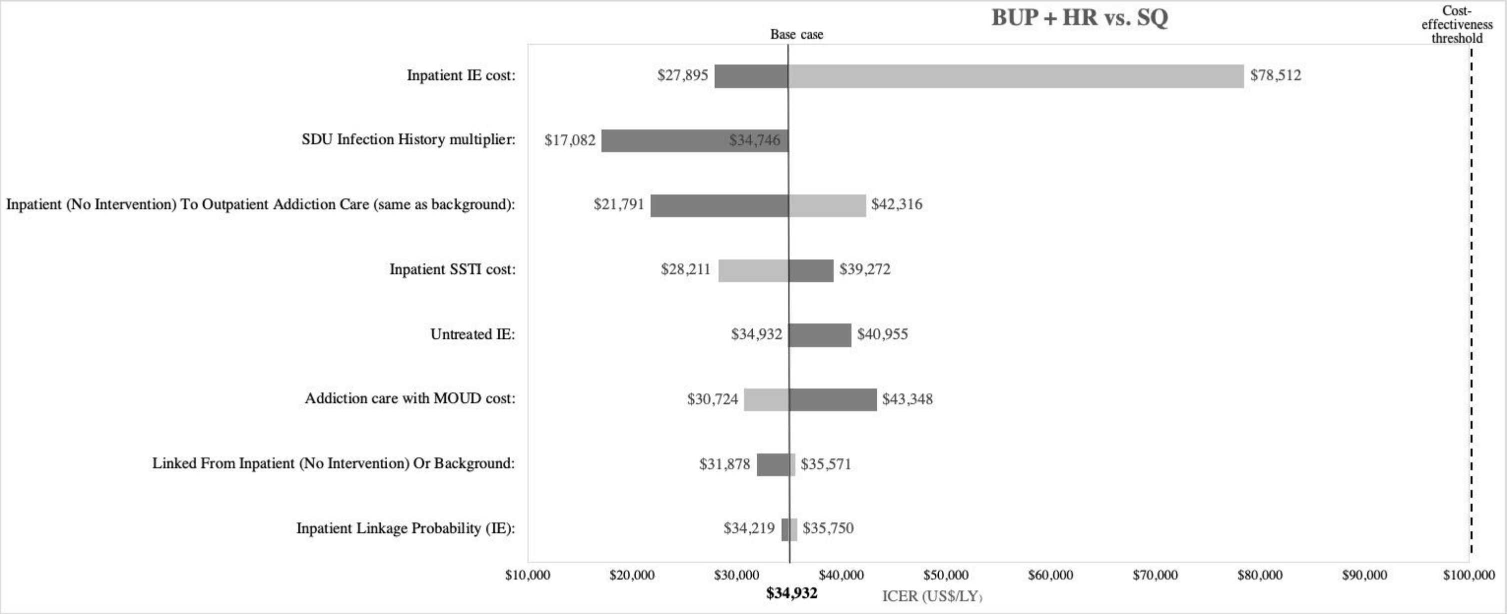

Abbreviations: BUP: Buprenorphine; HR: Harm Reduction; ICER: incremental cost-effectiveness ratio; LY: life years; IE: infective endocarditis; SDU: sequelae of drug use; SSTI: severe skin and soft tissue infections; MOUD: medication for opioid use disorder.

The tornado diagram shows the full ICER range when a parameter value in the model is varied from its lowest to highest bounds while keeping the other parameter values constant. This tornado diagram shows the ICERs from the combined BUP+HR strategy compared with the status quo. The dark grey bars represent the effect of the highest bounds and the light grey bar represents the effect of the lowest bound on costs. The bars are centered around the base case ICER of $34,932 to provide a reference for the changes. The dashed line represents the cost-effectiveness threshold of $100,000.

© 2023 Jawa R et al. *JAMA Network Open.*
