# Supplementary material for: Estimated Clinical Outcomes and Cost-effectiveness Associated With Provision of Addiction Treatment in US Primary Care Clinics
Source: JAMA Netw Open. 2023 Apr 12;6(4):e237888. doi: 10.1001/jamanetworkopen.2023.7888 (PMC10098970; doi:10.1001/jamanetworkopen.2023.7888)
Supplement: Supplement 2. — Data Sharing Statement [file jamanetwopen-e237888-s002.pdf]

Data Sharing Statement

Jawa. Estimated Clinical Outcomes and Cost-effectiveness Associated With Provision of Addiction Treatment in US Primary Care Clinics. *JAMA Netw Open*. Published April 12, 2023. doi:10.1001/jamanetworkopen.2023.7888

Data
Data available: No

Additional Information
Explanation for why data not available: We developed a decision analytic model. There is no patient data or a data dictionary.
